# Intravital imaging of splenic classical monocytes modifying the hepatic CX3CR1^+^ cells motility to exacerbate liver fibrosis via spleen-liver axis

**DOI:** 10.7150/thno.87791

**Published:** 2024-03-03

**Authors:** Chenlu Han, Yujie Zhai, Yuke Wang, Xuwen Peng, Xian Zhang, Bolei Dai, Yuehong Leng, Zhihong Zhang, Shuhong Qi

**Affiliations:** 1 Britton Chance Center and MoE Key Laboratory for Biomedical Photonics, Wuhan National Laboratory for Optoelectronics-Huazhong University of Science and Technology, Wuhan, Hubei 430074, China.; 2 State key laboratory of digital medical engineering, School of Biomedical Engineering, Hainan University, Haikou, Hainan 570228, China.

**Keywords:** liver fibrosis, hepatic and splenic CX3CR1^+^ cells, spatial localization, movement behavior, spleen-liver axis

## Abstract

CX3CR1^+^ cells play a crucial role in liver fibrosis progression. However, changes in the migratory behavior and spatial distribution of spleen-derived and hepatic CX3CR1^+^ cells in the fibrotic liver as well as their influence on the liver fibrosis remain unclear.

Methods: The CX3CR1^GFP/+^ transgenic mice and CX3CR1-KikGR transgenic mice were used to establish the CCl4-induced liver fibrosis model. Splenectomy, adoptive transfusion of splenocytes, *in vivo* photoconversion of splenic CX3CR1^+^ cells and intravital imaging were performed to study the spatial distribution, migration and movement behavior, and regulatory function of CX3CR1^+^ cells in liver fibrosis.

Results: Intravital imaging revealed that the CX3CR1^GFP^ cells accumulated into the fibrotic liver and tended to accumulate towards the central vein (CV) in the hepatic lobules. Two subtypes of hepatic CX3CR1^+^ cells existed in the fibrotic liver. The first subtype was the interacting CX3CR1^GFP^ cells, most of which were observed to distribute in the liver parenchyma and had a higher process velocity; the second subtype was mobile CX3CR1^GFP^ cells, most of which were present in the hepatic vessels with a faster moving speed. Splenectomy ameliorated liver fibrosis and decreased the number of CX3CR1^+^ cells in the fibrotic liver. Moreover, splenectomy rearranged CX3CR1^GFP^ cells to the boundary of the hepatic lobule, reduced the process velocity of interacting CX3CR1^GFP^ cells and decreased the number and mobility of mobile CX3CR1^GFP^ cells in the fibrotic liver. Transfusion of spleen-derived classical monocytes increased the process velocity and mobility of hepatic endogenous CX3CR1^GFP^ cells and facilitated liver fibrosis progression via the production of proinflammatory and profibrotic cytokines. The photoconverted splenic CX3CR1^+^ KikRed^+^ cells were observed to leave the spleen, accumulate into the fibrotic liver and contact with hepatic CX3CR1^+^ KikGreen^+^ cells during hepatic fibrosis.

Conclusion: The splenic CX3CR1^+^ monocytes with classical phenotype migrated from the spleen to the fibrotic liver, modifying the migratory behavior of hepatic endogenous CX3CR1^GFP^ cells and exacerbating liver fibrosis via the secretion of cytokines. This study reveals that splenic CX3CR1^+^ classical monocytes are a key driver of liver fibrosis via the spleen-liver axis and may be potential candidate targets for the treatment of chronic liver fibrosis.

## Introduction

Liver fibrosis is a chronic and inflammatory procedure characterized by excessive accumulation of extracellular matrix, with potentially life-threatening complications 1. Leukocytes (neutrophils, DCs, macrophages, monocytes and T cells) residing in or infiltrating the liver participated in the pathogenesis of liver fibrosis 2-4. Recent evidence suggests that spleen-derived immune cells participate in the progression of liver fibrosis 5, 6, while splenectomy has been proven to alleviate liver fibrosis 7, 8, suggesting that the spleen-liver axis may play a crucial role in chronic hepatic diseases. However, the underlying regulatory mechanisms of the involvement of the spleen-liver axis, especially spleen-derived immune cells (such as splenic monocytes), in liver fibrosis remain unclear.

The spleen serves as an important monocyte reservoir under inflammatory conditions 5, 9-17, which participates in the liver fibrosis procession. It has been reported that the spleen acts as a major source of circulating monocytes with a high potential of migration to injury sites 18, 19. Chemokine (C-X3-C motif) ligand 1 (CX3CL1) and its receptor CX3CR1 play a crucial role in regulating the inflammatory response. The CX3CL1-CX3CR1 interaction alleviates liver fibrosis, but CX3CR1 deficiency exacerbated the progression of liver fibrosis and increased monocyte/macrophage infiltration into the fibrotic liver 20, 21. CX3CR1 expression has been reported on circulating monocytes, hepatic macrophages, dendritic cells, etc 22, 23. Growing evidence indicated that CX3CR1^+^ monocyte/macrophages (Mon/Mφs) aggravated the inflammatory response and exacerbated fibrosis by promoting myofibroblasts accumulation 24-28. A recent study showed that a subtype of CD11b^+^ CD43^high^ Ly6C^low^ splenic monocytes migrated into the liver and acquired macrophage features to exacerbate liver fibrosis 5. Nevertheless, the possible involvement of splenic CX3CR1^+^ monocytes in the development of liver fibrosis remains uncertain.

The migration of immune cells between the organs was usually studied by using photoconvertible fluorescence protein transgenic mice. The previous research studied photoconverted splenocytes migrating from the spleen into the fibrotic liver by using KikGR transgenic mice 5. Intravital imaging is a powerful tool for real-time visualization of the spatial distribution, movement behavior and function of immune cells in liver diseases. *In vivo* photoacoustic and fluorescence imaging revealed that the distribution of KC along the central vein (CV)-portal triad (PT) axis followed a linear pattern in each liver lobule 29. Whole-mount three-dimensional imaging showed that rearrangement of CD11c^+^ DCs at the portal lobular boundary in the early stages of fibrotic liver might enhance hepatic inflammation and fibrogenesis 30. Furthermore, intravital imaging showed the restricted movement of Cytotoxic T lymphocytes (CTLs) in liver metastasis lesions, indicating that CTLs recognize and interact with tumor cells to trigger tumor cell death 31. Therefore, to explain the modulation role splenic CX3CR1^+^ cells play in the progression of hepatic fibrosis, it is necessary to track the migration of splenic CX3CR1^+^ monocytes and study the spatial distribution and movement behavior of CX3CR1^+^ cells in the fibrotic liver.

In this study, by using CX3CR1^GFP/+^ transgenic mice with CCl4-induced liver fibrosis combined with intravital imaging, the spatial distribution and the movement behavior of hepatic CX3CR1^GFP^ cells in the fibrotic liver were explored. Intravital imaging revealed the accumulation of CX3CR1^GFP^ cells into the fibrotic liver, which tended to accumulate towards the central vein in the hepatic lobules. In the fibrotic liver, interacting CX3CR1^GFP^ cells in the liver parenchyma had a higher process velocity and mobile CX3CR1^GFP^ cells in the hepatic vessels had a faster moving speed. Splenectomy shifted the CX3CR1^GFP^ cells distribution toward the boundary of the hepatic lobule, reduced the process velocity of interacting CX3CR1^GFP^ cells and decreased the number and migratory velocity of mobile CX3CR1^GFP^ cells in the fibrotic liver. Splenic CX3CR1^+^ KikRed^+^ monocytes were visualized to migrate from the host spleen and preferentially recruit into the fibrotic liver. Transfusion of splenic CX3CR1^+^ cells from fibrotic mice did not cause the spatial rearrangement of hepatic endogenous CX3CR1^GFP^ cells, but significantly altered their migratory behavior. Moreover, splenic CX3CR1^+^ cells (especially the classical monocytes subset) were proven to exacerbate the fibrotic procession by secreting pro-inflammatory and profibrotic cytokines. These findings provide new insights into the role of splenic CX3CR1^+^ subset cells in fibrotic progression through the spleen-liver axis, and are valuable for developing a CX3CR1^+^ subset cells-targeted therapy for liver fibrosis.

## Results

### CX3CR1^+^ cells significantly expanded in the fibrotic liver and splenectomy caused a decrease in the number of CX3CR1^+^ monocyte/macrophages (Mon/Mφs)

**Figure 1:**
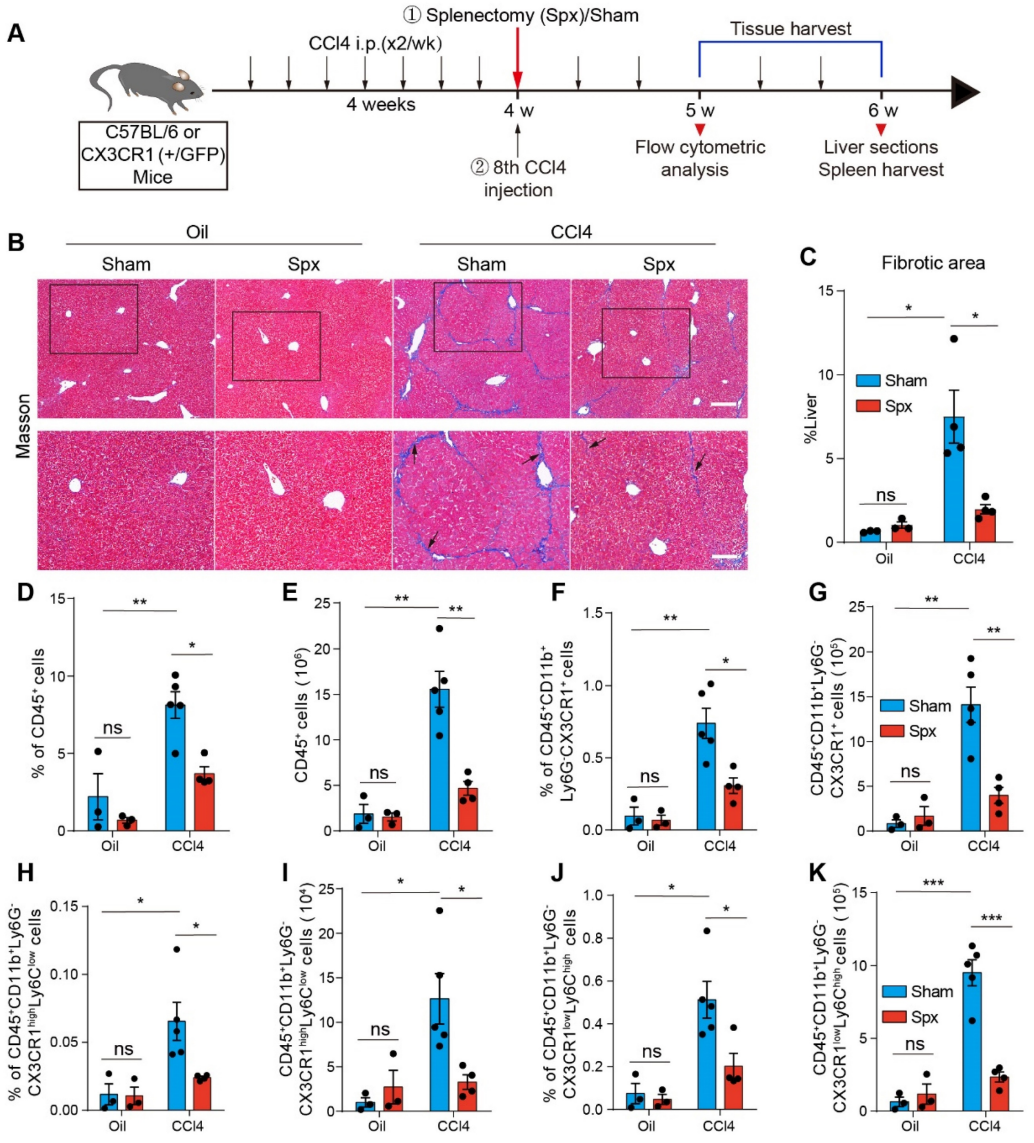
** The effect of splenectomy on the percentage and number of hepatic CX3CR1^+^ cells and their subsets.** (**A**) Schedules of generation of the liver fibrosis model and splenectomy. (**B**) Liver sections were stained with Masson trichrome. Black arrows indicate collagen positive areas. Top row: Large-field images. Scale bar, 200 µm. Bottom row: Images of the regions of interest from the top row. Scale bar, 100 µm. (**C**) Image‐based quantification of collagen positive area in liver sections (n = 3-4 mice per group). (**D-E**) The percentage and number of hepatic CD45^+^ cells in Oil-treated mice or CCl4-treated mice at one week after splenectomy (n = 3-5 mice per group). (**F-G**) The percentage and number of hepatic CD45^+^ CD11b^+^ Ly6G^-^ CX3CR1^+^ cells in Oil-treated mice or CCl4-treated mice at one week after splenectomy (n = 3-5 mice per group). (**H-I**) The percentage and number of hepatic CD45^+^ CD11b^+^ Ly6G^-^ CX3CR1^high^ Ly6C^low^ cells in Oil-treated mice or CCl4-treated mice at one week after splenectomy (n = 3-5 mice per group). (**J-K**) The percentage and number of hepatic CD45^+^ CD11b^+^ Ly6G^-^ CX3CR1^low^ Ly6C^high^ cells in Oil-treated mice or CCl4-treated mice at one week after splenectomy (n = 3-5 mice per group). Data are presented as the mean ± SEM.

We generated a murine liver fibrosis model with repeated intraperitoneal injections of CCl4 (1 mL/kg, twice a week, and Coin Oil as control) for 6 weeks (Figure 1A). After 6 weeks, the spleen body ratios of CCl4-treated (fibrotic) mice were 1.54-fold higher than those of the Oil-treated (control) mice (Figure S1A and S1B). Compared with Oil-treated mice, CCl4-induced liver fibrosis significantly increased the percentage (3.18% to 6.18%) (Figure S1C) and the number of CX3CR1^+^ cells (1.38×10^6^ to 4.57×10^6^) in the spleen of mice (Figure S1D). Flow cytometry analysis showed that the CX3CR1^+^ cells from the fibrotic spleen expressed high levels of CD45, CD11b, CD68, Ly6C and medium levels of F4/80, CD11c, MHC-Ⅱ and low levels of Ly6G, CD4, CD8, B220, CD19 (Figure S1E), indicating that most of CX3CR1^+^ cells exhibited phenotypic characteristics of monocytes/macrophages (96.93% CD68, 59.77% Ly6C and 14.50% F4/80) in the fibrotic spleen. We also found that there was no marked difference in the phenotypic characteristics of splenic CX3CR1^+^ cells between Oil-treated group and CCl4-treated group (Figure S1E).

Splenectomy has been proven to alleviate liver fibrosis 5, 16. Thus, we performed splenectomy (Spx) or sham surgery (Sham) before the 8th Oil/CCl4 injection (Figure 1A). Hepatic collagen deposition was detected by Masson trichrome staining. Compared with control mice, CCl4-treated mice showed prominent liver fibrosis with increased collagen deposition, whereas splenectomy markedly reduced liver fibrosis (Figure 1B and 1C). To determine the functional contribution of splenic monocytes to liver fibrosis progression, we performed splenectomy on the CX3CR1^GFP/+^ transgenic mice, in which most monocytes and macrophages express the green fluorescent protein (GFP) 32. We analyzed the percentage and number of hepatic CD45^+^ cells and CX3CR1^+^ cells one week after splenectomy by flow cytometry. The gating strategy was shown in Figure S2A-B. The data showed that splenectomy did not affect the percentage and number of CD45^+^ cells (Figure 1D and 1E) and CD45^+^ CD11b^+^ Ly6G^-^ CX3CR1^+^ cells (Figure 1F and 1G) in the livers of Oil-treated mice. Compared with Oil-treated mice, the percentage and number of hepatic CD45^+^ cells (Figure 1D and 1E) and CD45^+^ CD11b^+^ Ly6G^-^ CX3CR1^+^ cells (Figure 1F and 1G) significantly increased in the livers of CCl4-treated mice. Splenectomy significantly decreased the percentage and number of CD45^+^ cells (Figure 1D and 1E) and CD45^+^ CD11b^+^ Ly6G^-^ CX3CR1^+^ cells (Figure 1F and 1G) in the livers of CCl4-treated mice. These results indicated that the spleen might be an essential reservoir for CX3CR1^+^ cells in liver fibrosis.

Splenic monocytes can be classified into classical and non-classical monocytes 19, 33. Classical monocytes express high levels of Ly6C and low levels of the fractalkine receptor CX3CR1. Non-classical monocytes express low levels of Ly6C and high levels of CX3CR1 34. We analyzed the effect of CCl4-induced liver fibrosis and splenectomy on the percentage and number of hepatic classical (CD45^+^ CD11b^+^ Ly6G^-^ CX3CR1^low^ Ly6C^high^) Mon/Mφs and non-classical (CD45^+^ CD11b^+^ Ly6G^-^ CX3CR1^high^ Ly6C^low^) Mon/Mφs by flow cytometry. The gating strategy was shown in Figure S2B. Compared with Oil-treated mice, CCl4-induced liver fibrosis significantly increased the percentage and number of hepatic non-classical Mon/Mφs (Figure 1H and 1I) and classical Mon/Mφs (Figure 1J and 1K) in the liver of mice. Splenectomy significantly decreased the percentage and number of non-classical Mon/Mφs (Figure 1H and 1I) and classical Mon/Mφs (Figure 1J and 1K) in the fibrotic liver. Most of CX3CR1^+^ cells exhibited phenotypic characteristics of monocytes/macrophages (CD68^+^, F4/80^+^, CD11c^+^ and Ly6C^+^) in the fibrotic liver (Figure S3A). Flow cytometry analysis showed that splenectomy did not significantly alter the phenotypic characterization (CD68^+^, F4/80^+^ and CD11c^+^) (Figure S3B-D) of hepatic CD45^+^ CD11b^+^ Ly6G^-^ CX3CR1^+^ cells, hepatic CX3CR1^+^ non-classical and classical Mon/Mφs in CCl4-treated mice (Figure S3E-G). Splenectomy obviously reduced the abundance of CX3CR1^+^ non-classical and classical Mon/Mφs in the CCl4-induced fibrotic liver, suggesting that the subsets of CX3CR1^+^ Mon/Mφs in the fibrotic liver probably were derived from the spleen.

### Splenectomy changed the spatial distribution of CX3CR1^GFP^ cells in the fibrotic liver lobes

**Figure 2:**
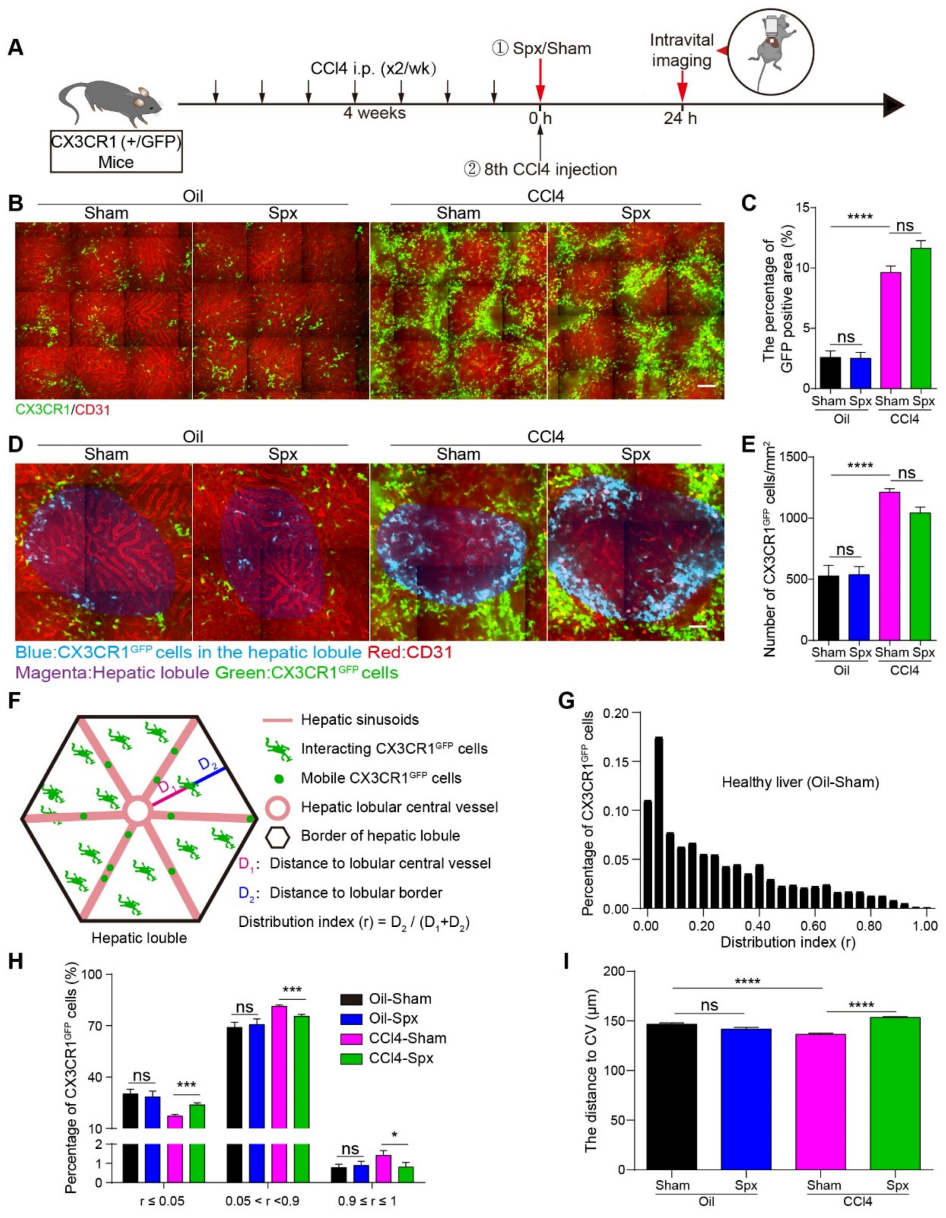
** Intravital imaging of the spatial distribution and localization of CX3CR1^GFP^ cells in liver lobule after splenectomy.** (**A**) Schedules of generation of liver fibrosis model, the splenectomy and intravital imaging. (**B**) Intravital imaging of CX3CR1^GFP^ cells distribution in the liver. Green: CX3CR1^GFP^ cells; Red: AF (Alexa Fluor) 647 anti-CD31 labeled hepatic vessels. Scale bar, 100 µm. (**C**) The positive areas of GFP from three random views of each mouse were quantified using Image J software (n = 9, from 3 mice per group). (**D**) High-magnification views of the CX3CR1^GFP^ cells distribution in the hepatic lobule. Blue: CX3CR1^GFP^ cells in the hepatic lobule; Red: AF647 anti-CD31 labeled hepatic vessels; Magenta: Hepatic lobule; Green: CX3CR1^GFP^ cells. Scale bar, 50 µm. (**E**) The density of CX3CR1^GFP^ cells in one hepatic lobule (n = 12-18). (**F**) Schematic diagram of the distribution of CX3CR1^GFP^ cells in hepatic lobules. (**G**) The distribution of CX3CR1^GFP^ cells in each distribution index (r) from the healthy liver of Oil-treated group with Sham; the bin (a bin is an interval into which a given set of data is divided) value is 0.04. (H) The percentages of CX3CRl^GFP^ cells in the different ranges of distribution index; the bin value is 0.005. (**I**) The distance of CX3CR1^GFP^ cells to the hepatic central vein (CV) in the hepatic lobules. Data are presented as mean ± SEM.

The functions of Mon/Mφs in the liver are closely related to their spatial localization 29, 30. The hepatic lobule is the basic structural and functional unit of the liver 35, 36. Therefore, studying the arrangement of CX3CR1^GFP^ cells in the hepatic lobules of the fibrotic liver is valuable. We performed intravital imaging on the exposed livers of different CX3CR1^GFP/+^ transgenic mice groups 24 h after the 8th CCl4 injection (Oil-treated group with Spx, Oil-treated group with Sham, CCl4-treated group with Spx, and CCl4-treated group with Sham, Figure 2A).

Compared with the Oil-treated group, CCl4-treatment markedly increased the density of CX3CR1^GFP^ cells in the liver (Figure 2B). The percentage of GFP-positive area in the liver was 3.73-fold higher in the CCl4-treated group than that in the Oil-treated group (Figure 2C). Additionally, the density of CX3CR1^GFP^ cells in one hepatic lobule was 2.22-fold higher in the CCl4-treated group than that in the Oil-treated group (Figure 2D and 2E). Within 24 h, splenectomy did not significantly change the percentage of GFP-positive area and the density of CX3CR1^GFP^ cells in one hepatic lobule in either the Oil-treated group or the CCl4-treated group (Figure 2C and 2E), indicating that splenectomy did not affect the percentage and number of CX3CR1^GFP^ cells in a short time, probably due to the complex hepatic immune microenvironment.

To precisely characterize the spatial localization and distribution of CX3CR1^GFP^ cells in the hepatic lobules, we defined the distribution index r = *D*2 / (*D*1 + *D*2) as described in previous research 30. The distribution index (r) ranges from 0 to 1, r ≤ 0.05 indicates that the cells are close to the boundary of the hepatic lobules and 0.9 ≤ r ≤ 1 indicates that the cells are distributed surrounding the central vein (CV) (Figure 2F). Intravital imaging data indicated that the distribution index of CX3CR1^GFP^ cells from different groups was consistent with a lognormal distribution in the hepatic lobules (Figure 2G and S4A-C).

In the hepatic lobules of Oil-treated mice, 1.00% of CX3CR1^GFP^ cells were distributed surrounding the CV (0.9 ≤ r ≤ 1), and 28.96% of CX3CR1^GFP^ cells were distributed in the marginal region of hepatic lobular (r ≤ 0.05). 24 h after splenectomy, 0.90% of CX3CR1^GFP^ cells were distributed surrounding the CV (0.9 ≤ r ≤1), and 28.46% of CX3CR1^GFP^ cells were distributed in the marginal region of the hepatic lobule (r ≤ 0.05) (Figure 2H). These data indicated that splenectomy did not significantly alter the spatial localization and distribution of CX3CR1^GFP^ cells in Oil-treated mice. In CCl4-treated mice, the percentage of CX3CR1^GFP^ cells distributing surrounding the CV (0.9 ≤ r ≤ 1) was 1.42%, and then decreased to 0.80% 24 h after splenectomy. The percentage of CX3CR1^GFP^ cells located at the hepatic lobular boundary (r ≤ 0.05) was 17.28% in CCl4-treated mice, and then increased to 23.72% after splenectomy (Figure 2H). These results suggested that splenectomy did not significantly change the spatial distribution of CX3CR1^GFP^ cells in the Oil-treated mice, but obviously modified their spatial localization and distribution in the hepatic lobules of the fibrotic liver.

Next, we calculated the distribution distance of CX3CR1^GFP^ cells to the CV in the hepatic lobules. The average distance of CX3CR1^GFP^ cells to the CV in the hepatic lobules of Oil-treated mice was 143.62 ± 1.36 µm. After splenectomy, the average distance was 141.81 ± 1.69 µm, indicating that splenectomy did not significantly influence the distribution distance of CX3CR1^GFP^ cells to the CV. The average distance of CX3CR1^GFP^ cells to the CV in the hepatic lobules of CCl4-treated mice decreased to 136.41 ± 1.17 µm, whereas splenectomy increased the average distance to 153.35 ± 0.95 µm in the hepatic lobules (Figure 2I). These results suggested that CX3CR1^GFP^ cells accumulated toward the CV in the hepatic lobules of the fibrotic liver and splenectomy skewed their accumulation toward the hepatic lobular boundary.

These results indicated that the spatial localization of CX3CR1^GFP^ cells in the hepatic lobules is closely related to fibrosis progression. The unique localized pattern of CX3CR1^+^ cells toward the CV may help to spread more of CX3CR1^+^ cells-associated liver injury throughout the hepatic lobules and may greatly increase the contacts of CX3CR1^+^ cells with hepatic stellate cells in the different regions of hepatic lobules.

### Splenectomy changed the migratory behavior of CX3CR1^GFP^ cells in the fibrotic liver

**Figure 3:**
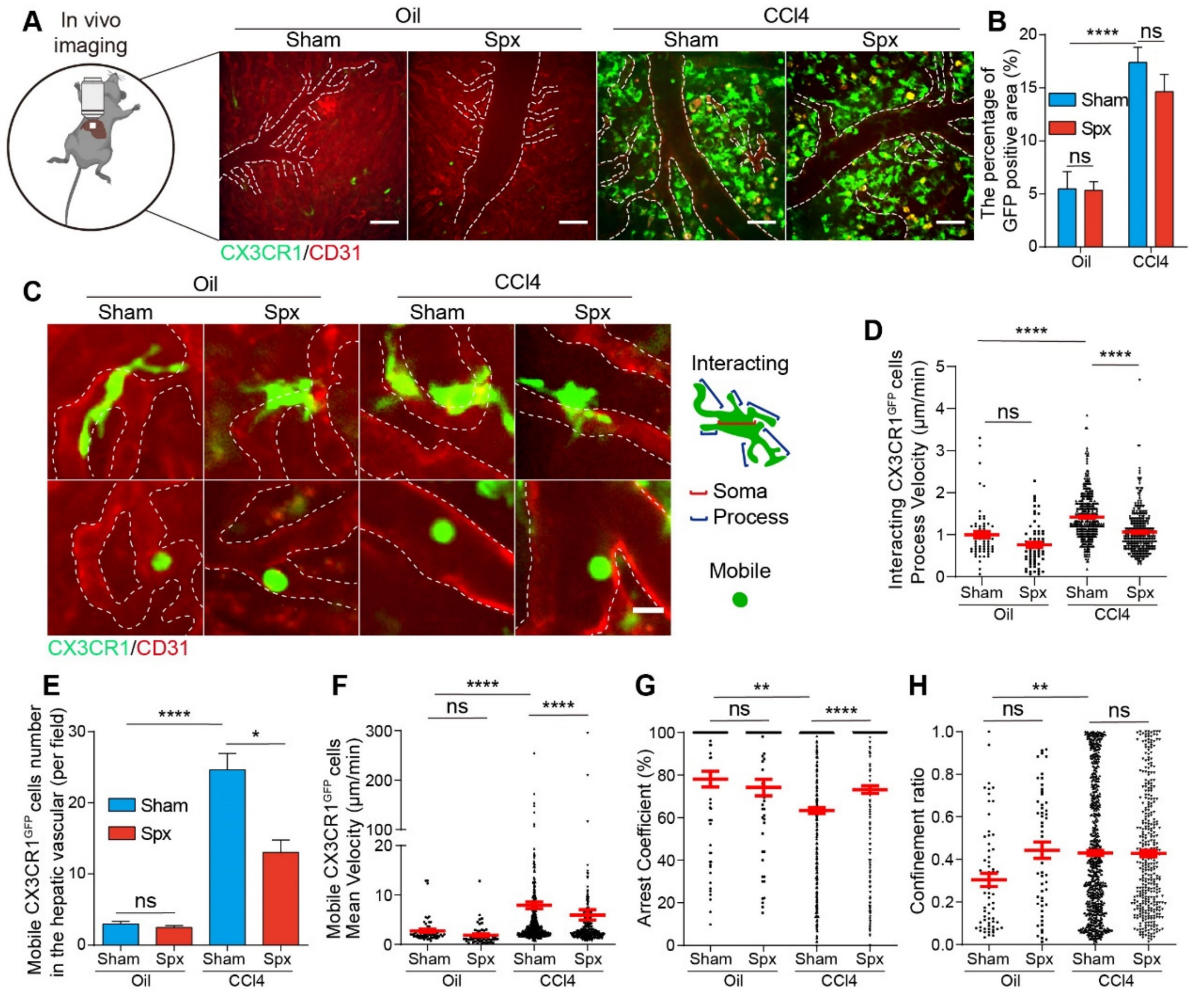
** Intravital imaging of CX3CR1^GFP^ cells in the livers of Oil/CCl4-treated mice with or without splenectomy at 24 h after splenectomy.** (**A**) Representative fluorescence images of the CX3CR1^GFP^ cells in the livers of Oil/CCl4-treated mice with or without splenectomy. Green: CX3CR1^GFP^ cells; Red: AF647 anti-CD31 labeled hepatic vessels. White dotted line shows the vessels. Scale bar, 50 μm. (**B**) The positive areas of GFP from three random views of each mouse were quantified using Image J software (n = 9, from 3 mice per group). (**C**) Subtypes of CX3CR1^GFP^ cells with differences in cell shape and distribution at 24h after splenectomy. White dotted line shows the vessels. Scale bar, 10 μm. (**D**) The velocity of interacting CX3CR1^GFP^ cells processes in the liver parenchyma. Each dot represents a single cell process, and the red bars indicate mean values (3 mice per group). (**E**) The cell number of mobile CX3CR1^GFP^ cells in the hepatic vessels of Oil/CCl4-treated mice with or without splenectomy (n = 22-28 fields, from 3 mice per group). (**F-H**) Scatter plots of mean velocity (**F**), arrest coefficient (**G**), and confinement ratio (**H**) of mobile CX3CR1^GFP^ cells in the hepatic vessels of Oil/CCl4-treated mice with or without splenectomy (n = 22-28 fields, from 3 mice per group). Each dot represents a single cell, and the red bars indicate mean values. Data are presented as mean ± SEM.

The function of CX3CR1^+^ cells in the liver is closely related to their migratory behavior. In order to study the migratory behavior of hepatic CX3CR1^GFP^ cells, we performed intravital imaging on the exposed livers of four groups (Oil-treated group with Spx or Sham, CCl4-treated group with Spx or Sham) 24 h after the 8th CCl4 injection (Figure 3A and S5A, Movie S1 and S2). The percentage of GFP-positive area in the liver was 3.18-fold higher in the CCl4-treated group than that in the Oil-treated group (Figure 3B). Within 24 h, splenectomy did not significantly change the percentage of GFP-positive area in the liver of either the Oil-treated group or the CCl4-treated group (Figure 3B). Previous work have shown that the morphology dynamics of Mon/Mφs are associated with their polarization 37 and activation 38. In this study, hepatic CX3CR1^GFP^ cells can be divided into two subtypes (interacting type and mobile type) according to their morphology and motility (Figure 3C), which was similar to the microglia types in the glioma 39, 40. The first cell type (interacting type) present in the liver parenchyma had a motile cell membrane with fast moving (extending and retracting) processes to contact with each other, and their cell soma did not show a significant movement (Figure 3C and S5B). The second cell type (mobile type) was almost all present in the hepatic vessels with a fast-moving motion and had an amoeboid shape without any processes (Figure 3C and S5C).

The motility of two subtypes of CX3CR1^GFP^ cells in four groups was analyzed by dynamic quantitative parameters, such as the mean velocity, arrest coefficient and confinement ratio 41, 42. The mean velocity represents the migratory speed of CX3CR1^GFP^ cells; the arrest coefficient represents the percentage of time that each CX3CR1^GFP^ cell remained arrested; the confinement ratio represents the ratio of the displacement of each CX3CR1^GFP^ cell to its total length within a given time.

As the interacting type of CX3CR1^GFP^ cells showed no obvious movement of their cell soma in the liver parenchyma, we focused on the migratory behavior of their processes and tracked individual ending of the process of interacting CX3CR1^GFP^ cell from its starting position to its final position over time in two dimensions (Figure S5D). Intravital imaging data showed that the interacting type of CX3CR1^GFP^ cells had a significantly higher process velocity in CCl4-treated group than Oil-treated group (1.42 ± 0.03 μm/min in CCl4-treated group, and 1.00 ± 0.08 μm/min in Oil-treated group), indicating that interacting CX3CR1^GFP^ cells increased their crosstalk with each other in the fibrotic liver. We also found that splenectomy significantly decreased the mean process velocity of interacting CX3CR1^GFP^ cells (from 1.42 ± 0.03 μm/min to 1.06 ± 0.03 μm/min) (Figure 3D) and shortened the process trajectories (Figure S5E) of interacting CX3CR1^GFP^ cells in the liver of CCl4-treated group, while there was no marked difference in the process velocity of interacting CX3CR1^GFP^ cells between Oil-treated group with Spx (0.76 ± 0.06 μm/min) and Sham (1.00 ± 0.08 μm/min) (Figure 3D). Taken together, these results indicated that splenectomy primarily decreased the contact and crosstalk between interacting CX3CR1^GFP^ cells in the CCl4-induced fibrotic liver, but had no effect on the contact between interacting CX3CR1^GFP^ cells in the healthy liver of Oil-treated group.

Next, we would like to study the migratory behavior of the mobile type of CX3CR1^GFP^ cells in the hepatic vessels and track the center of individual mobile CX3CR1^GFP^ cell from its starting position to its final position over time in two dimensions (Figure S5F). As shown in Figure 3E, splenectomy did not affect the number of mobile CX3CR1^GFP^ cells in the healthy liver of Oil-treated group, but it significantly reduced the number of mobile CX3CR1^GFP^ cells in the fibrotic liver of CCl4-treated group. Compared with the Oil-treated group, mobile CX3CR1^GFP^ cells in the fibrotic liver of CCl4-treated group displayed broader trajectories with a higher mean velocity and lower arrest coefficient (mean velocity: 7.93 ± 0.70 μm/min in CCl4-treated group versus 2.76 ± 0.31 μm/min in Oil-treated group, and arrest coefficient: 0.63 ± 0.14 in CCl4-treated group versus 0.78 ± 0.04 in Oil-treated group, respectively) (Figure 3F-H and S5G). Splenectomy did not significantly change the migratory behavior of mobile CX3CR1^GFP^ cells in the healthy liver of Oil-treated group, but it significantly shortened the trajectories, decreased the velocity of mobile CX3CR1^GFP^ cells and increased their arrest coefficient in the fibrotic liver of CCl4-treated group (5.97 ± 1.06 μm/min and 0.73 ± 0.18 in the CCl4-treated group with Spx, versus 7.93 ± 0.70 μm/min and 0.63 ± 0.14 in CCl4-treated group with Sham) (Figure 3F-H and S5G). Splenectomy decreased the process velocity of interacting CX3CR1^GFP^ cells and attenuated the number and migratory function of mobile CX3CR1^GFP^ cells in the fibrotic liver, possibly leading to the inhibition of CX3CR1^GFP^ cells recruitment, decreased intercellular communications.

The mobile type of CX3CR1^GFP^ cells in the hepatic vessels could be divided into three types of interactions according to their motility, which referred to the mobile types of the tumor infiltrating lymphocytes (TILs) in CFP-B16 tumor 43. These three mobile types are “stable” type (mean velocity < 2 μm/min, arrested and closely contact with vessel endothelial cells), “confined” type (mean velocity: 2-3 μm/min, not completely arrested but move around vessel endothelial cells) and “serial” type (mean velocity > 3 μm/min, interact with vessel endothelial cells transiently or not interact with any vessel endothelial cells) (Figure S6A-C). The observed stable and confined interactions between mobile CX3CR1^GFP^ cells and vessel endothelial cells indicated that these mobile CX3CR1^GFP^ cells might sense the inflammatory chemokines in the liver, bind to the vessel endothelial cells. In the CCl4-treated group, 20.64% of mobile CX3CR1^GFP^ cells were in a stable state, 26.65% were confined, and 52.71% were in a serial state (Figure S6D). This result suggested that almost half (20.64% “stable” type and 26.65% “confined” type) of mobile CX3CR1^GFP^ cells remained stable or confined interactions with vessel endothelial cells. We also found that the number of the mobile CX3CR1^GFP^ cells in a stable (5.09 cells/field) or confined (6.57 cells/field) state in the CCl4-treated group (total 11.66 cells/field) was significantly higher than those (total 2.40 cells/field,1.57 cells/field in a stable state and 0.83 cells/field in a confined state) in the Oil-treated group (Figure S6E). This result suggested that marked increases in the numbers of the “stable” and “confined” type of CX3CR1^GFP^ cells in fibrotic liver (Figure S6E), which might contribute to the enhancement of mobile CX3CR1^GFP^ cells infiltration into the fibrotic liver and the exaggeration of liver inflammation and fibrosis.

The different profiles of markers expression on hepatic mobile and interacting CX3CR1^GFP^ cells were analyzed by the flow cytometry (Figure S7A-C). For the interacting CX3CR1^GFP^ cells presented in the liver parenchyma and mobile CX3CR1^GFP^ cells almost presented in the hepatic vessels. Thus, we extracted peripheral blood cells from mice with liver fibrosis to study the phenotypic characteristics of mobile CX3CR1^GFP^ cells, and isolated non-parenchymal cells from the fibrotic liver that perfused via the portal vein with PBS to study the phenotypic characteristics of interacting CX3CR1^GFP^ cells. The flow cytometry analysis showed that most of mobile CX3CR1^GFP^ cells (93.63%) exhibited the characteristics of monocytes/macrophages (CD68, Ly6C and F4/80), and a small portion (<10%) might be DCs, B cells, etc (Figure S7A and S7C). And most of interacting CX3CR1^GFP^ cells (97.87%) were monocytes/macrophages (CD68, Ly6C and F4/80), while a small part (<10%) might be DCs, T cells, B cells, etc (Figure S7B and S7C). Although the cell surface marker profile of mobile CX3CR1^GFP^ cells was similar to the interacting CX3CR1^GFP^ cells, the interacting CX3CR1^GFP^ cells with a much mature status (F4/80-positive interacting and mobile CX3CR1^GFP^ cells: 52.47% versus 12.09%) and higher antigen-presenting ability (MHC-II-positive interacting and mobile CX3CR1^GFP^ cells: 47.20% versus 7.99%) in the fibrotic liver (Figure S7C). Moreover, the flow cytometry analysis also showed that the splenectomy did not significantly alter the surface marker profile of mobile and interacting CX3CR1^GFP^ cells (Figure S7C).

Next, the effects of the timing of splenectomy on the migratory behavior of CX3CR1^GFP^ cells in the fibrotic liver were also analyzed (Figure S8A-J). We performed intravital imaging on the exposed livers at 14 days after splenectomy (6 weeks for induction of liver fibrosis) in the fibrotic mice (Figure S8A-B, Movie S3). The result showed that splenectomy decreased the percentage of GFP-positive area of CX3CR1^GFP^ cells in the fibrotic liver (Figure S8C), but had no effect on the process velocity and trajectories of interacting CX3CR1^GFP^ cells in the fibrotic liver (Sham: 0.95 ± 0.04 μm/min; Spx: 0.85 ± 0.04 μm/min) (Figure S8D and S8I). Additionally, splenectomy did not significantly change the number of mobile CX3CR1^GFP^ cells in the fibrotic liver (Figure S8E). There was no marked difference in the migratory behavior (Figure S8F-H) and trajectories (Figure S8J) of mobile CX3CR1^GFP^ cells between CCl4-treated group with Spx and Sham (mean velocity: Sham: 6.09 ± 0.40 μm/min; Spx: 5.95 ± 0.34 μm/min, and arrest coefficient: Sham: 0.63 ± 0.02; Spx: 0.61 ± 0.02). These findings indicated that at 14 days after splenectomy, it did not affect the migratory behavior of interacting CX3CR1^GFP^ cells and mobile CX3CR1^GFP^ cells, possibly due to the steady state of the immune system.

### Splenic CX3CR1^+^ cells (classical monocytes) of fibrotic mice facilitated liver fibrosis progression

**Figure 4:**
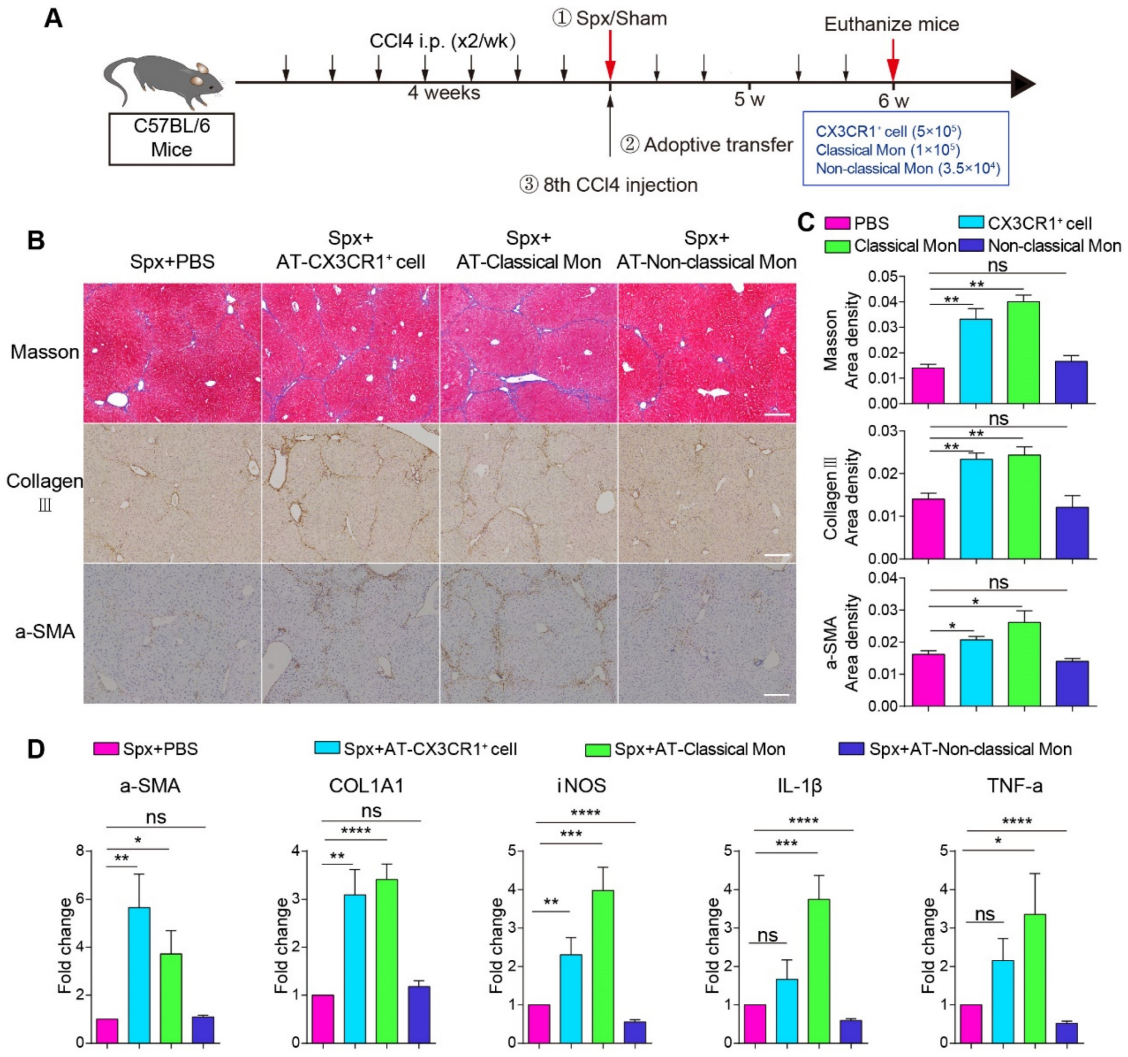
**The pro-fibrotic effects of splenic classical monocytes isolated from fibrotic mice.** (**A**) Schedules of the splenectomy and the adoptive transfer experiments. (**B**) Liver sections were stained with Masson trichrome, collagen III, alpha-smooth muscle actin (α-SMA). Scale bar, 200 μm. (**C**) The proportion of positive areas in liver sections was quantified (4-5 mice per group). (**D**) The expression levels of fibrosis-associated genes alpha-SMA (α-SMA), Collagen type I alpha1 (COL1A1) and proinflammatory genes iNOS, IL-1β and TNF-a in the livers of different groups were measured using qPCR. Data represent relative mRNA levels to an endogenous internal standard (beta-actin mRNA). Data are presented as mean ± SEM.

To identify which subtype of splenic monocytes plays a key role in promoting hepatic fibrosis, we adoptively transferred splenic CX3CR1^+^ cells, classical monocytes and non-classical monocytes from fibrotic spleen into the CCl4-treated mice with splenectomy. We analyzed the percentages of splenic CD11b^+^ CX3CR1^+^ cells and their subsets among splenocytes in the fibrotic spleen (CD11b^+^ CX3CR1^+^ cells: 1.15%, classical monocytes: 0.26%, non-classical monocytes: 0.09%) by flow cytometry (Figure S9A-B). The number of adoptive splenic monocytes were determined according to the previous literature 13, 44 and flow cytometry data. The CX3CR1^GFP/+^ fibrotic mice were sacrificed before the 8th CCl4 injection and the splenic CD11b^+^ CX3CR1^+^ cells (5×10^5^, named AT-CX3CR1^+^ cells group), classical monocytes (CD11b^+^ CD115^+^ CX3CR1^low^ Ly6C^high^, 1×10^5^, named AT-classical Mon group) and non-classical monocytes (CD11b^+^ CD115^+^ CX3CR1^high^ Ly6C^low^, 3.50×10^4^, named AT-non-classical Mon group) were isolated from CX3CR1^GFP/+^ fibrotic mice by flow cytometric cell sorting (Figure S9A). The purity of sorted cells was more than 90% (Figure S9C and S9D). Then, the mice were sacrificed 14 days after adoptive transfer (Figure 4A).

The phenotypic characteristics of adoptive splenic CX3CR1^+^ cells were also analyzed by immunofluorescence staining and flow cytometry. Immunofluorescence data showed that CX3CR1^+^ cells in the marginal zone and subcapsular red pulp of the fibrotic spleen expressed CD68 and F4/80, indicating these cells display monocytic phenotype (Figure S9E). The phenotypic characteristics (CD68 and F4/80) of splenic CX3CR1^+^ cells and the subsets (CD11b^+^ CD115^+^ CX3CR1^low^ Ly6C^high^ classical monocytes and CD11b^+^ CD115^+^ CX3CR1^high^ Ly6C^low^ non-classical monocytes) were further analyzed by flow cytometry (Figure S9A). The expression of cell surface molecules in the two splenic CX3CR1^+^ cell subsets differed slightly between Oil-treated group and CCl4-treated group (Figure S10A-E), indicating CCl4 treatment did not significantly affect the expression of cell surface molecules in the two splenic CX3CR1^+^ cell subsets.

Next, we wanted to evaluate the effect of adoptive splenic cells on hepatic fibrosis progression. Hepatic collagen deposition was detected by Masson trichrome 16 and collagen III staining 5. The activation of hepatic stellate cells was detected by alpha-smooth muscle actin (α-SMA) staining 5, 45. Compared with splenectomy mice, adoptive transfusion of monocytes or classical monocytes, but not non-classical monocytes, significantly exacerbated liver fibrosis with increased collagen deposition and up-regulation of hepatic stellate cells (HSCs) activation (Figure 4B and 4C). The transfusion of CD11b^+^ CX3CR1^+^ cells or classical monocytes, but not non-classical monocytes, significantly increased the expression of fibrosis-associated genes α-SMA (5.65- and 3.72-fold, respectively) and COL1A1 (3.09- and 3.41-fold, respectively) in the liver compared with splenectomy mice. Furthermore, the transfusion of classical monocytes obviously increased the expression of pro-inflammation genes iNOS (3.98-fold), IL-1β (3.75-fold) and TNF-a (3.35-fold) in the liver (Figure 4D). These data suggested that splenic monocytes and splenic classical monocytes isolated from the fibrotic mice, might be a key driving force of hepatic fibrosis progression and exacerbate liver fibrosis via secreting pro-inflammatory and profibrotic cytokines.

### Splenic CX3CR1^+^ classical monocytes from fibrotic mice affected the spatial distribution and migratory behavior of hepatic CX3CR1^GFP^ cells

**Figure 5:**
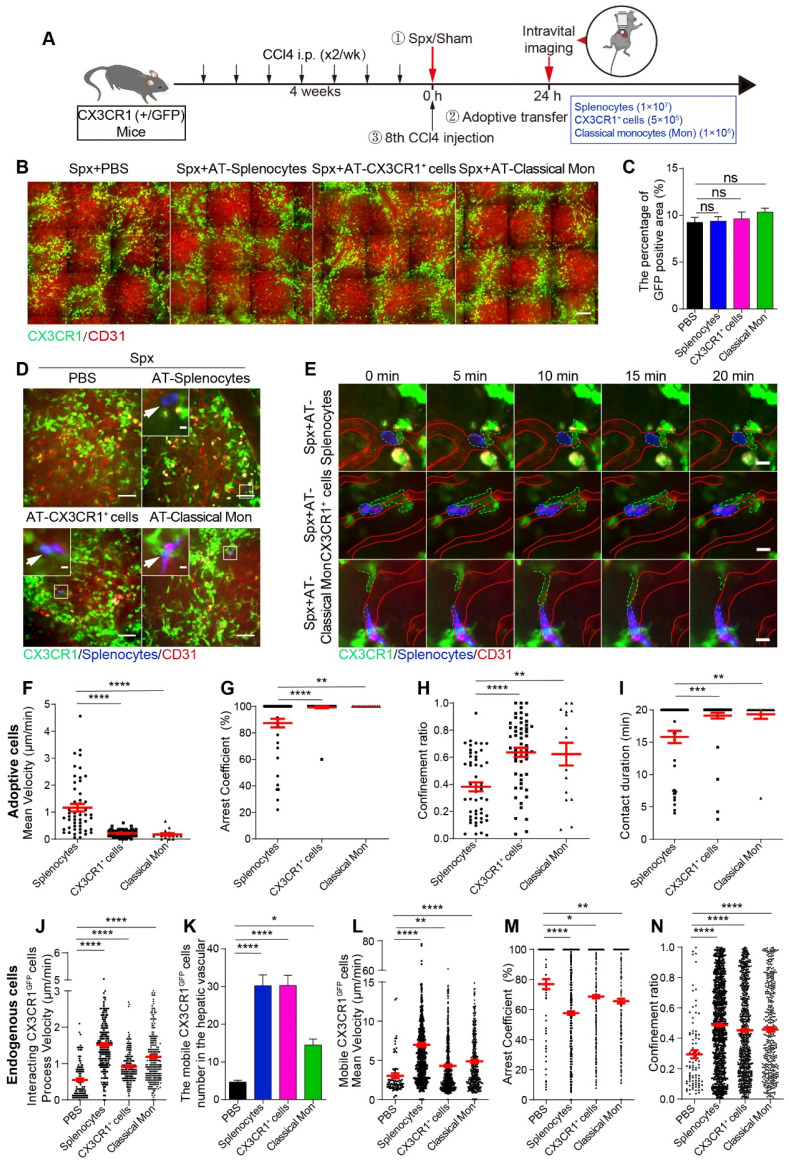
Intravital imaging of **spatial distribution and migration behavior of endogenous CX3CR1^GFP^ cells and adoptive splenic cells in the fibrotic liver.** (**A**) Schedules of the splenectomy, the adoptive transfer and intravital imaging experiments. (**B**) Intravital imaging of CX3CR1^GFP^ cells distribution in the liver. Green: CX3CR1^GFP^ cells; Red: AF647 anti-CD31 labeled hepatic vessels. Scale bar, 100 µm. (**C**) The positive areas of GFP from three random views of each mouse were quantified using Image J software (n = 9, from 3 mice per group). (**D**) Intravital imaging of adoptive splenic cells in the liver. Red: AF647 anti-CD31 labeled hepatic vessels; Blue: Splenocytes; Green: CX3CR1^GFP^ cells. Scale bar (large image), 50 μm. Scale bar (small image), 5 µm. White arrows indicate the adoptive splenic cells. (**E**) Intravital imaging of the dynamic interaction between adoptive splenic cells and hepatic CX3CR1^GFP^ cells. Red: AF647 anti-CD31 labeled hepatic vessels; Blue: Splenocytes; Green: CX3CR1^GFP^ cells. Scale bar, 10 μm. (**F-H**) Scatter plots of mean velocity (**F**), arrest coefficient (**G**), and confinement ratio (**H**) of adoptive splenic cells in different groups (3 mice per group). Each dot represents a single cell, and the red bars indicate mean values. (**I**) The duration of contact between adoptive cells and hepatic CX3CR1^GFP^ cells. Each dot represents a single cell, and the red bars indicate mean values. The data are presented as the mean ± SEM (3 mice per group). (**J**) The velocity of interacting CX3CR1^GFP^ cells processes in the liver parenchyma (3 mice per group). Each dot represents a single cell process, and the red bars indicate mean values. (**K**) The cell number of mobile CX3CR1^GFP^ cells in the hepatic vessels of different groups. The data are presented as the mean ± SEM (n = 19-28 fields, from 3 mice per group). (**L-N**) Scatter plots of mean velocity (**L**), arrest coefficient (**M**), and confinement ratio (**N**) of mobile CX3CR1^GFP^ cells in the hepatic vessels of different groups. Each dot represents a single cell, and the red bars indicate mean values. Data are presented as mean ± SEM.

Next, we would like to evaluate the roles of splenic cells in the migratory behavior and spatial distribution changes of hepatic endogenous CX3CR1^GFP^ cells during liver fibrosis. TdTomato-positive (tdT^+^) splenic cells (1×10^7^, named AT-splenocytes group) were isolated from ROSA^mT/mG^ mice with liver fibrosis, which express strong red fluorescence protein tdTomato in cell types 46. The ROSA^mT/mG^ mice were sacrificed before the 8th CCl4 injection. Meanwhile, splenic CD11b^+^ CX3CR1^+^ cells (5×10^5^, named AT-CX3CR1^+^ cells group) and classical monocytes (CD11b^+^ CD115^+^ CX3CR1^low^ Ly6C^high^, 1×10^5^, named AT-classical Mon group) were isolated from CX3CR1^GFP/+^ fibrotic mice by flow cytometric cell sorting (Figure S9A and S9B), and the purity of sorted cells was more than 90% (Figure S9C and S9D). After splenectomy, tdT^+^ splenic cells, red fluorescent dye (Cell Tracker Red, CMPTX) labeled CX3CR1^+^ cells and classical monocytes were transferred into CCl4-treated CX3CR1^GFP/+^ transgenic mice prior to the 8th CCl4 injection. 24 h later, we performed intravital imaging on the exposed livers (Figure 5A).

The effect of adoptive splenic cells on the spatial distribution and localization of endogenous CX3CR1^GFP^ cells in the fibrotic liver was evaluated. Intravital imaging showed that the transfusion of splenic cells did not alter the percentage of GFP-positive area (Figure 5B and 5C) and the density of CX3CR1^GFP^ cells in one hepatic lobule in the splenectomy group (Figure S11A-B). Moreover, the distribution index of the CX3CR1^GFP^ cells in the splenectomy group and adoptive transfer groups was consistent with a lognormal distribution in the hepatic lobules (Figure S11C-F). There was no significant difference in the distribution index of CX3CR1^GFP^ cells (Figure S11G) and the average distance from CX3CR1^GFP^ cells to CV (Figure S11H) between the splenectomy group and adoptive transfer groups. These results indicated that transferring adoptive splenic cells did not significantly affect the spatial distribution of CX3CR1^GFP^ cells within 24 h after splenectomy.

Intravital imaging revealed that adoptive splenocytes reached the fibrotic liver within 24 h after transfusion (Figure 5D and S12A, Movie S4 and S5). The adoptive cells were distributed randomly in the hepatic vessels (Figure 5D-E, S12A-C and S13A, Movie S4-7). The long-time interactions between hepatic endogenous CX3CR1^GFP^ cells and adoptive cells existed not only in the AT-splenocytes group but also in the AT-CX3CR1^+^ cells and AT-classical Mon groups. Interestingly, intravital imaging and 3D immunofluorescence imaging (21 μm on the Z axis) investigated that some endogenous CX3CR1^GFP^ cells in the liver parenchyma penetrated their motile processes into the hepatic vessels to form stable contacts with adoptive splenic cells (Figure 5D-E and S12A-C, Movie S4-7), indicating that the crosstalk might exist between hepatic endogenous CX3CR1^GFP^ cells and adoptive splenic cells.

The migratory behavior and trajectories (Figure S13B) of adoptive cells in the liver were analyzed. When compared with adoptive splenocytes, adoptive splenic CX3CR1^+^ cells and classical monocytes showed slower speed (mean velocity: 0.21 ± 0.02 μm/min in AT-CX3CR1^+^ cells and 0.17 ± 0.04 μm/min in AT-classical Mon; versus 1.17 ± 0.14 μm/min in AT-splenocytes, respectively) (Figure 5F), more arrest (arrest coefficient: 0.99 ± 0.01 in AT-CX3CR1^+^ cells and 1.00 ± 0.00 in AT-classical Mon; versus 0.87 ± 0.03 in AT-splenocytes, respectively) (Figure 5G), and shortened displacement (confinement ratio: 0.64 ± 0.03 in AT-CX3CR1^+^ cells and 0.62 ± 0.08 in AT-classical Mon; versus 0.38 ± 0.03 in AT-splenocytes, respectively) (Figure 5H and S13B), displaying a longer duration of contact with hepatic CX3CR1^GFP^ cells (durations: 19.12 ± 0.46 in AT-CX3CR1^+^ cells and 19.32 ± 0.68 in AT-classical Mon; versus 15.82 ± 0.94 in AT-splenocytes, respectively) (Figure 5I). These results indicated that adoptive CX3CR1^+^ cells and the adoptive classical monocytes displayed similar migratory behavior and could form long-time and stable contacts with hepatic endogenous CX3CR1^+^ cells, which might be important for the onset of inflammatory responses. The splenic CX3CR1^+^ cells and classical monocytes likely played a key role in promoting liver fibrosis progression.

To evaluate the phenotypic characteristics of neighboring endogenous CX3CR1^GFP^ cells in contact with adoptive splenic monocytes in the liver (Figure S14A-C), the splenic cells (1×10^7^, named AT-healthy splenocytes group) from CX3CR1^GFP/+^ healthy mice were adoptively transferred into fibrotic mice with splenectomy (Figure S14A) as described previously. Compared with adoptive splenocytes from healthy mice, adoptive splenic CX3CR1^+^ cells and classical monocytes from fibrotic mice contacted with neighboring endogenous CX3CR1^GFP^ cells (GFP) highly expressed M1 phenotypic markers CD68 (AT-CX3CR1^+^ cells: 77.78% and AT-classical Mon: 76.47% versus AT-healthy splenocytes: 55.56%), F4/80 (81.82% and 90.91% versus 58.82%), CD86 (77.78% and 80.00% versus 55.56%), but lowly expressed M2 phenotypic marker CD206 (33.33% and 30.00% versus 60.00%) (Figure S14D). These results indicated that adoptive splenic CX3CR1^+^ cells and classical monocytes from fibrotic mice were more likely to interact with pro-inflammatory M1-CX3CR1^+^ macrophages in the liver, which might drive liver fibrosis progression.

Next, we focused on the effect of adoptive cells on the migratory behavior of endogenous CX3CR1^GFP^ cells in the liver. Compared with splenectomy group, endogenous interacting CX3CR1^GFP^ cells in the AT-splenocytes group, AT-CX3CR1^+^ cells group and AT-classical Mon group had a significantly higher process velocity (1.51 ± 0.05 μm/min in AT-splenocytes mice, 0.92 ± 0.03 μm/min in AT-CX3CR1^+^ cells mice, 1.19 ± 0.05 μm/min in AT-classical Mon mice; versus 0.55 ± 0.04 μm/min in CCl4-treated with Spx mice) (Figure 5J) and displayed broader process trajectories (Figure S15A, Movie S4 and S5), indicating that transfusion of splenocytes, CX3CR1^+^ cells and classical monocytes significantly increased the crosstalk among endogenous interacting CX3CR1^GFP^ cells, possibly modulating pro-fibrogenic response.

Besides, we observed increased numbers of mobile CX3CR1^GFP^ cells in the AT-splenocytes, AT-CX3CR1^+^ cells and AT-classical Mon group compared with the splenectomy group (Figure 5K). The trajectories of mobile CX3CR1^GFP^ cells in adoptive transfer groups were obviously broader than those in the splenectomy group (Figure S15B, Movie S4 and S5). Mobile CX3CR1^GFP^ cells in the AT-splenocytes group displayed a higher mean velocity and a lower arrest coefficient compared with the splenectomy group (6.96 ± 0.27 μm/min and 0.58 ± 0.01 in AT-splenocytes group; versus 3.03 ± 0.32 μm/min and 0.77 ± 0.03 in splenectomy group, respectively) (Figure 5L-N). The transfusion of CX3CR1^+^ cells and classical monocytes also increased the motility of mobile CX3CR1^GFP^ cells in splenectomy mice, demonstrating an increased mean velocity and a decreased arrest coefficient (4.32 ± 0.16 μm/min and 0.69 ± 0.01 in AT-CX3CR1^+^ cells group, 4.89 ± 0.20 μm/min and 0.66 ± 0.02 in AT-classical Mon group; versus 3.03 ± 0.32 μm/min and 0.77 ± 0.03 in splenectomy group) (Figure 5L-N). The migratory behavior of mobile CX3CR1^GFP^ cells in the AT-CX3CR1^+^ cells and AT-classical Mon group was similar to that in the AT-splenocytes group, indicating that transfusion of splenocytes, especially splenic CX3CR1^+^ cells and classical monocytes, increased the number and moving speed of mobile CX3CR1^GFP^ cells in the hepatic vessels, possibly promoting CX3CR1^GFP^ cells recruitment and driving pro-fibrogenic response.

Next, we compared the migratory behavior of endogenous mobile CX3CR1^GFP^ cells and adoptive splenic cells (Figure S16A-I). Compared with adoptive splenic cells, hepatic endogenous mobile CX3CR1^GFP^ cells showed broader trajectories (Figure S13B and S15B) with a higher mean velocity (6.96 ± 0.27 μm/min in endogenous mobile CX3CR1^GFP^ cells, versus 1.17 ± 0.14 μm/min in AT-splenocytes; 4.32 ± 0.16 μm/min in endogenous mobile CX3CR1^GFP^ cells, versus 0.21 ± 0.02 μm/min in AT-CX3CR1^+^ cells; 4.89 ± 0.20 μm/min in endogenous mobile CX3CR1^GFP^ cells, versus 0.17 ± 0.04 μm/min in AT-classical Mon) (Figure S16A-C) and lower arrest coefficient (0.58 ± 0.01 in endogenous mobile CX3CR1^GFP^ cells, versus 0.87 ± 0.03 in AT-splenocytes; 0.69 ± 0.01 in endogenous mobile CX3CR1^GFP^ cells, versus 0.99 ± 0.01 in AT-CX3CR1^+^ cells; 0.66 ± 0.02 in endogenous mobile CX3CR1^GFP^ cells, versus 1.00 ± 0.00 in AT-classical Mon) (Figure S16D-F). These results demonstrated that adoptive splenic CX3CR1^+^ cells showed slower speed and little net displacement, whereas hepatic endogenous mobile CX3CR1^+^ cells had a higher migration speed. These data indicated that the CX3CR1^+^ cells derived from different organs exhibited different migratory properties and played distinct roles in the progression of liver fibrosis.

### Splenic CX3CR1^+^ cells were visualized to migrate from the spleen and accumulate into the fibrotic liver *in vivo*

**Figure 6:**
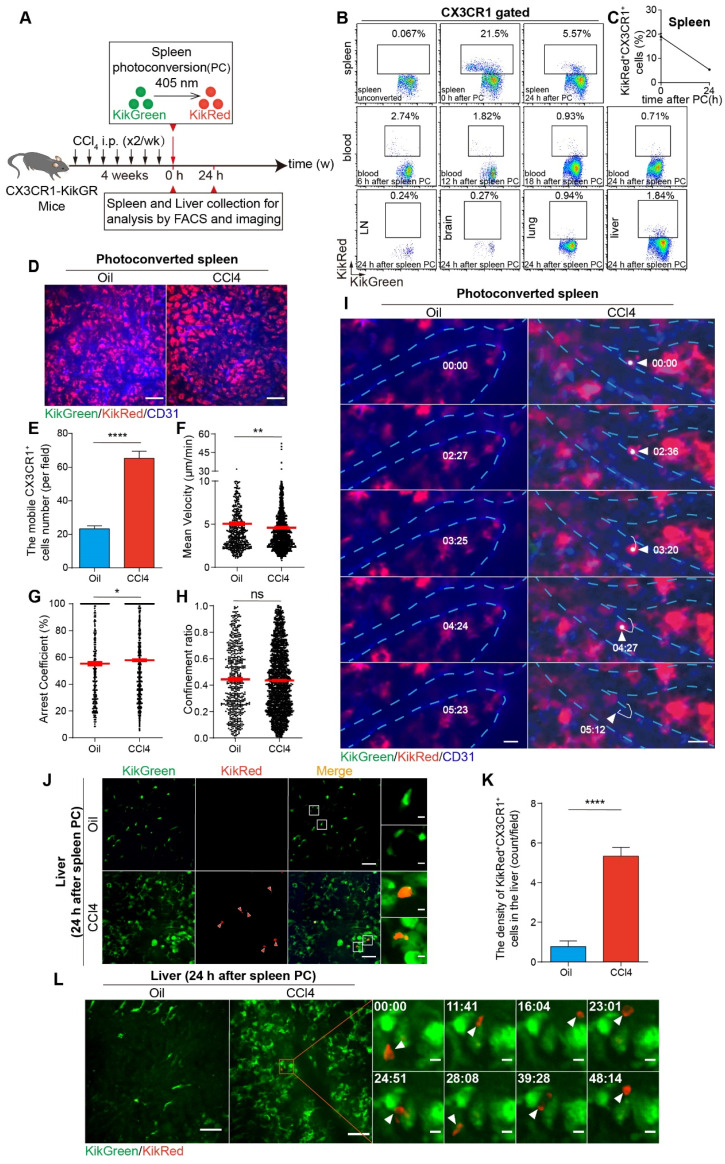
** Flow cytometry analysis and intravital imaging of the photoconverted splenic CX3CR1^+^ cells in the spleen and fibrotic liver.** (**A**) Schedules of photoconversion experiments, the intravital imaging and flow cytometry. (**B**) Representative flow cytometry plots showing the egress of photoconverted CX3CR1^+^ KikRed^+^ cells from the fibrotic spleen and their redistribution to different organs (peripheral blood, lymph node, brain, lung and liver) over a 24-hour period (n = 3-5 mice per group). (**C**) The percentage of the photoconverted CX3CR1^+^ KikRed^+^ cells among CX3CR1^+^ cells in the spleen over a 24-hour period (n = 4 mice per group). (**D**) Intravital imaging of photoconverted spleen of Oil and CCl4-treated mice. Scale bar, 50 μm. (**E**) The cell number of CX3CR1^+^ cells in the photoconverted spleen from Oil and CCl4-treated mice (n = 23-24 fields, from 3 mice per group). (**F-H**) Scatter plots of mean velocity (**F**), arrest coefficient (**G**), and confinement ratio (**H**) of CX3CR1^+^ cells in the photoconverted spleen from Oil and CCl4-treated mice. Each dot represents a single cell, and the red bars indicate mean values. (**I**) Intravital imaging of CX3CR1^+^ KikRed^+^ cells in the spleen of Oil and CCl4-treated mice. Green: KikGreen; Red: KikRed; Blue: AF647 anti-CD31 labeled splenic vessels. Scale bar, 10 μm. White arrows indicate a splenic CX3CR1^+^ KikRed^+^ cell. (**J**) Intravital imaging of splenic CX3CR1^+^ KikRed^+^ cells in fibrotic livers of mice that received spleen-specific photoconversion. Scale bar, 50 μm. High-magnification views of splenic CX3CR1^+^ KikRed^+^ cells in the fibrotic liver are shown on the right. Scale bar, 5 µm. Red arrows indicate KikRed^+^ cells. (**K**) The splenic CX3CR1^+^ KikRed^+^ cells in livers were counted (cells/field). (n = 9 fields, from 3 mice per group). (**L**) Intravital imaging of the liver of Oil and CCl4-treated mice 24 h after spleen-specific photoconversion. Green: KikGreen; Red: KikRed. Scale bar, 50 μm. High-magnification views of splenic CX3CR1^+^ KikRed^+^ cells in the fibrotic liver are shown on the right. Scale bar, 5 µm. White arrows indicate splenic CX3CR1^+^ KikRed^+^ cells. Data are presented as mean ± SEM.

To verify whether splenic CX3CR1^+^ cells migrate into the fibrotic liver *in vivo*, we used CX3CR1-KikGR transgenic mice in which CX3CR1^+^ cells expressed photoconvertible fluorescent protein Kikume Green-Red (KikGR), which can be converted from green to red following exposure to the 405 nm light. The spleen cells were photoconverted by exposure to the 405 nm light (three sides, each side was exposed for 3 minutes, 200 mW/cm^2^) prior to the 8th CCl4 injection (Figure 6A and S17A). The procedure of CX3CR1-KikGR photoconversion in spleen did not affect the viability of splenocytes within 24 h (viability of unconverted and photoconverted splenocytes-0 h: 86.27% and 84.33%; viability of unconverted and photoconverted splenocytes-24 h: 83.10% and 83.97%) (Figure S17B). Imaging analysis revealed that the KikRed^+^ fluorescence signal persisted for 144 h and KikRed protein levels remained stable in photoconverted cells (relative percentage of KikRed^+^ fluorescence-0 h: 99.66%, 24 h: 98.98%, 48 h: 75.62%, 72 h: 59.79% and 144 h: 23.83%) for the duration of the experiments (Figure S17C-E). Then, the proportion of photoconverted CX3CR1^+^ cells in the organs of CCl4-treated mice were analyzed by flow cytometry (Figure 6B and S18A-B). The flow cytometry data showed that before photoconversion, the percentage of CX3CR1^+^ KikRed^+^ cells among splenic CX3CR1^+^ cells was only 0.067%. After photoconversion, it increased to 18.98%, and then decreased to 5.37% 24 h later (Figure 6B-C). We also observed a decrease in the number of photoconverted splenic CX3CR1^+^ KikRed^+^ cells (4.06×10^4^: from 7.16×10^4^ to 3.10×10^4^) 24 h after photoconversion (Figure S18C). To identify the organs to which the majority of the splenic CX3CR1^+^ KikRed^+^ cells migrated, we traced splenic CX3CR1^+^ KikRed^+^ cells redistribution throughout the body over a 24-hour period using flow cytometry. 6 h after photoconversion, we observed an increased distribution of CX3CR1^+^ KikRed^+^ cells in peripheral blood (Figure 6B and S18D). 24 h after photoconversion, the CX3CR1^+^ KikRed^+^ cells appeared in the fibrotic liver, and their percentage among CX3CR1^+^ cells in the liver increased to 1.71% (Figure 6B and S18E). Besides, we observed that the percentage of CX3CR1^+^ KikRed^+^ cells among CX3CR1^+^ cells in the fibrotic liver were much higher than those in the other organs (lymph node: 0.23%, brain: 0.14%, lung: 0.81% and peripheral blood: 0.98%) (Figure S18E). We also estimated the percentage and number of photoconverted splenic CX3CR1^+^ KikRed^+^ cells in different organs of fibrotic mice. Approximately 14% (∼0.57×10^4^) of photoconverted splenic CX3CR1^+^ KikRed^+^ cells (∼4.06×10^4^) migrated into the fibrotic liver, significantly higher than that migrated into other organs (lymph node: 0.04% and ∼0.18×10^2^; brain: 0.01% and ∼0.03×10^2^; lung: 4.98% and ∼0.20×10^4^; peripheral blood: 5.60% and ∼0.23×10^4^; versus liver: 13.98% and∼0.57×10^4^) (Figure S18F-G). The rest of the splenic CX3CR1^+^ KikRed^+^ cells might migrate to other organs, such as the lung, gut, kidney and heart. These data indicated that photoconverted CX3CR1^+^ cells egressed from the spleen, appeared in the blood and approximately 14% of them migrated to fibrotic liver.

Then, we wanted to study the migratory behaviors and trajectories (Figure S18H) of CX3CR1^+^ cells in the spleen. The spleen was exposed for photoconversion by 405 nm light and 0.5 h later intravital imaging was performed on the exposed spleen. Intravital imaging data revealed the complete and fast photoconversion of splenic CX3CR1^+^ cells from green to red fluorescence (Figure 6D, Movie S8). Compared with Oil-treated mice, the number of mobile CX3CR1^+^ cells in the spleen of CCl4-treated mice significantly increased (Figure 6E). Intravital imaging data showed that mobile CX3CR1^+^ cells displayed a lower mean velocity (4.59 ± 0.09 μm/min in CCl4-treated group versus 5.04 ± 0.15 μm/min in Oil-treated group) (Figure 6F) and a higher arrest coefficient (0.58 ± 0.01 in CCl4-treated group versus 0.55 ± 0.01 in Oil-treated group) in the CCl4-treated group compared with Oil-treated group (Figure 6G). There was no significant difference in the confinement ratio between Oil- and CCl4-treated groups (Figure 6H). These results suggested that CX3CR1^+^ cells exhibited reduced motility in the spleen of fibrotic mice. The whole process (for approximately 5 minutes) of a CX3CR1^+^ KikRed^+^ cell leaving the spleen of fibrotic mice was observed. In the fibrotic mice, CX3CR1^+^ KikRed^+^ cell crossed the vessel wall into the blood flow and then disappeared, which was not observed in Oil-treated mice (Figure 6I, Movie S9).

Finally, we analyzed the accumulation of CX3CR1^+^ KikRed^+^ cells into the fibrotic liver. Compared with Oil-treated mice, the density of splenic CX3CR1^+^ KikRed^+^ cells increased 6.86-fold in the livers of fibrotic mice (increase in the density of splenic CX3CR1^+^ KikRed^+^ cells from 0.78 cells/field to 5.33 cells/field) (Figure 6J-K). Intravital imaging showed that splenic CX3CR1^+^ KikRed^+^ cells frequently contacted with several hepatic CX3CR1^+^ KikGreen^+^ cells (Figure 6L, Movie S10) or formed stable interactions with neighboring CX3CR1^+^ KikGreen^+^ cells (Figure S18I, Movie S11) in the livers of fibrotic mice. To analyze the effect of the splenectomy on the accumulation of CX3CR1^+^ KikRed^+^ cells into the fibrotic liver, we performed splenectomy (Spx) or sham surgery (Sham) after photoconversion and conducted intravital imaging on the exposed livers at 24 h after splenectomy. Compared to CCl4-treated group with Sham, 24 h after splenectomy, the density of splenic CX3CR1^+^ KikRed^+^ cells were decreased by 83.33% in the livers of CCl4-treated group with Spx (decrease in the density of splenic CX3CR1^+^ KikRed^+^ cells from 6.67 cells/field to 1.11 cells/field) (Figure S18J-K). The imaging data indicated that the splenectomy reduced the accumulation of splenic CX3CR1^+^ KikRed^+^ cells in the fibrotic liver. These results revealed that CX3CR1^+^ KikRed^+^ cells with a constrained migration pattern left from the spleen by crossing the vessel wall into the blood and finally disappearing in a short time (for 5 minutes), then accumulated into the fibrotic liver.

## Discussion

Using intravital imaging, this study investigated the spatial and behavioral changes of CX3CR1^GFP^ cells in the fibrotic liver, and splenectomy significantly reversed these changes. Combining the use of photoconvertible splenic CX3CR1^+^ cells and adoptive splenocytes transfusion with intravital imaging, we visualized splenic CX3CR1^+^ KikRed^+^ cells migrating from the spleen, accumulating into the fibrotic liver and contacting with hepatic endogenous CX3CR1^+^ KikGreen^+^ cells. Transfer of splenic cells also promoted the recruitment of hepatic mobile CX3CR1^GFP^ cells, accelerated the migration of mobile CX3CR1^GFP^ cells in the hepatic vessels and increased the process velocity of interacting CX3CR1^GFP^ cells in the liver parenchyma. Furthermore, splenic CX3CR1^+^ classical monocytes (CD11b^+^ CD115^+^ CX3CR1^low^ Ly6C^high^) exacerbated liver fibrosis via secreting proinflammatory and profibrotic cytokines. Taken together, these data revealed the profibrotic effects of splenic CX3CR1^+^ classical monocytes and provide insights into the hepatic CX3CR1^GFP^ cells spatial distribution and motility behavior modified by the spleen-liver axis.

The spleen is one of the primary extramedullary lymphatic organs and is closely related to the liver via the portal vein system 47. Spleen-derived cytokines 48 and leukocytes 6 have been shown to promote fibrogenesis; however, splenectomy improved liver fibrosis and ameliorated portal hypertension 49, 50, implying that the spleen-liver axis mediates the progression of liver fibrosis. Previous studies have shown that macrophages are one of the major leukocytes involved in the process of fibrosis 4, 51 and CX3CR1^+^ Mon/Mφs have profibrotic effects 25 27. Moreover, the spleen can also function as an extra reservoir of monocytes that are readily released in inflammatory conditions 14, 52, and splenic monocytes are able to migrate into the liver through the modulation of CX3CL1-CX3CR1 and CCL2-CCR2 axis and participate in the progression of hepatic fibrosis 16, 20, 21, 53. In this study, the number of CX3CR1^+^ cells markedly increased in the CCl4-induced fibrotic liver (Figure 1G) and spleen (Figure S1D), whereas splenectomy alleviated liver fibrosis (Figure 1 B-C) and reduced the number of CX3CR1^+^ cells (Figure 1G) and their subsets (CX3CR1^+^ non-classical and classical Mon/Mφs) (Figure 1I and 1K). Splenectomy impairs the recruitment of CX3CR1^+^ cells to the fibrotic liver, uncovering the role of the spleen as a storage site of CX3CR1^+^ monocytes during liver fibrosis.

Splenectomy and adoptive splenic cells transfer allowed us to study the role of splenic CX3CR1^+^ cells and their subsets in the liver fibrosis. Monocytes are a heterogeneous population comprised of distinct subsets with different cell surface markers and functional characteristics 54. However, their unique functions remain elusive due to the high heterogeneity. The present study found that a subset of splenic monocytes with “nonclassical”-like phenotype (CD11b^+^ CD43^hi^ Ly6C^lo^ monocytes) migrated into the fibrotic liver and shifted to macrophage, promoting liver fibrogenesis by activation of the hepatic stellate cells (HSCs) 5. In this study, we reported that the splenic classical monocytes (CD11b^+^ CD115^+^ CX3CR1^low^ Ly6C^high^), but not non-classical monocytes (CD11b^+^ CD115^+^ CX3CR1^high^ Ly6C^low^) aggravated the hepatic fibrosis with increased collagen deposition and up-regulation of hepatic stellate cells (HSCs) activation by secreting pro-inflammatory and profibrotic cytokines (Figure 4B-D). These results suggest that splenic monocytes consist of different subsets with subset-specific functions and play complicated roles in the progression of liver fibrosis.

There was a growing recognition that behavioral changes of immune cells in the liver were also highly related to their regulatory function 41 55. In this study, we observed two subtypes of CX3CR1^GFP^ cells in the liver classified by their morphology and motility (Figure 3C and S5B-C). The first subtype was interacting CX3CR1^GFP^ cells, most of them (97.87%) exhibited the characteristics of monocytes/macrophages (CD68, Ly6C and F4/80) (Figure S7B-C), while a small part (<10%) might be DCs, T cells, B cells, etc 22, 23. The second subtype was mobile CX3CR1^GFP^ cells, most of them (93.63%) were monocytes/macrophages (CD68, Ly6C and F4/80) (Figure S7A and S7C), and a small portion (<10%) might be DCs, B cells, etc 22, 23. In the CCl4-induced fibrotic liver, sensing extracellular stimuli and responding to changes in the fibrotic liver microenvironment, interacting CX3CR1^GFP^ cells adopted higher process velocities to gain more information from other neighboring CX3CR1^GFP^ cells and the complex inflammatory microenvironment (Figure 3D and S5D-E). Meanwhile, mobile CX3CR1^GFP^ cells in the hepatic vessels displayed increased number and moving speed (Figure 3E-H and S5F-G), probably due to chemokine-mediated recruitment 56 of CX3CR1^GFP^ cells into the fibrotic liver and their active search for other cells or extracellular stimuli 57. The splenectomy reversed the dynamic behavior of two subtypes of CX3CR1^GFP^ cells in the CCl4-induced fibrotic liver (Figure 3) within 24 h, possibly inhibiting CX3CR1^GFP^ cells infiltration and cellular interactions-mediated inflammatory response. However, at 14 days after splenectomy, it did not affect the migratory behavior of interacting CX3CR1^GFP^ cells and mobile CX3CR1^GFP^ cells (Figure S8), possibly due to the steady state of the immune system. Our findings indicated that CX3CR1^GFP^ cells' behavior regulation at the early stage of splenectomy might play an important role in modifying the progression of liver fibrosis.

A key question is how hepatic CX3CR1^+^ cells change their behavior in response to splenic CX3CR1^+^ cells. Intravital imaging and 3D immunofluorescence imaging showed that adoptive splenic CX3CR1^+^ cells and classical monocytes appeared in hepatic vessels and formed stable interactions with hepatic endogenous CX3CR1^GFP^ cells within 24 h after transfusion (Figure 5D-E, 5I, S12A-C and S13A), further highlighting and providing direct evidence for the existence of cellular interactions in the spleen-liver axis. Imaging analysis revealed that when sensing splenic CX3CR1^+^ cells (especially splenic classical monocytes) appearing in the liver, hepatic endogenous interacting CX3CR1^GFP^ cells in the liver parenchyma adjusted their higher process velocity to contact with each other (Figure 5J), and the hepatic endogenous mobile CX3CR1^GFP^ cells in the hepatic vessels adopted high-speed movement to accumulate into the fibrotic liver and searched for more other cells to transmit inflammatory signals (Figure 5L-N). Changes in behavior of hepatic CX3CR1^+^ cells when sensing splenic CX3CR1^+^ cells appeared to be prominent features of the inflammatory response in liver fibrosis.

Analysis of the kinetics of adoptive splenocytes movement revealed that adoptive splenocytes displayed more than one type of behavior (contact or migratory), and might have distinct migration pattern in the fibrotic liver (Figure 5F-I and S13B). After contact with hepatic CX3CR1^GFP^ cells, adoptive splenic CX3CR1^+^ cells and CX3CR1^+^ classical monocytes adopted slower speed and shortened displacement (Figure 5F-H, S13B and S16A-I), which were important for trigging inflammatory responses and promoting liver fibrosis. Whereas hepatic endogenous mobile CX3CR1^GFP^ cells displayed broader trajectories with a higher mean velocity and lower arrest coefficient (Figure 5L-N, S15B and S16A-I), which facilitates pro-inflammatory signal transduction. Comparison of the migratory behavior of adoptive splenic CX3CR1^+^ cells and hepatic endogenous mobile CX3CR1^GFP^ cells revealed that CX3CR1^+^ cells derived from different sources exhibited different migration characteristics and played a unique role in promoting the progression of liver fibrosis. Given the above results, studying the morphological and behavioral changes of hepatic CX3CR1^GFP^ cells in response to the splenic CX3CR1^+^ classical monocytes could help us understand how the spleen-liver axis works in liver fibrosis.

Recent studies have shown that the spatial location of hepatic Mon/Mφs was closely related to their function 58-60. Although studies have demonstrated that the scar-associated macrophage subpopulation adopted pro-fibrogenic phenotype and is topographically localized in the fibrotic niche 61, a deeper understanding of CX3CR1^GFP^ cells distributions in liver lobules is needed to identify interactions among different immune cells that might modulate liver fibrosis progression. Intravital imaging data indicated that a significant increase in the infiltration of CX3CR1^GFP^ cells in the fibrotic liver (Figure 2B-E) and an accumulation of CX3CR1^GFP^ cells towards the CV in the hepatic lobules (Figure 2H-I). This unique localization pattern of CX3CR1^GFP^ cells towards the CV may be a warning indicator of fibrosis progression. However, after splenectomy, CX3CR1^GFP^ cells tended to accumulate towards the hepatic lobular boundary (Figure 2H-I), suggesting that splenectomy has somehow reversed the spatial localization changes of CX3CR1^GFP^ cells in liver fibrosis progression. To some extent, strategic rearrangement of CX3CR1^GFP^ cells in the fibrotic liver lobules is essential for estimating the progression of liver fibrosis. Our findings showed that adoptive splenic cells did not significantly alter the density and spatial distribution of hepatic CX3CR1^GFP^ cells in the fibrotic mice that received splenectomy within a short time (about 24 hours) (Figure 5B-C and S11A-H), indicating that the complex spatial distribution of hepatic CX3CR1^GFP^ cells within different regions of hepatic lobules may be regulated by gradient changes in nutrition, oxygen concentration, hormones 62, 63 and chemokines-chemotactic cytokines 64-67 and other aspects.

KikGR as a photoconvertible Green-to-Red fluorescent protein has been used to label and track cell migration in different disease models, such as spondylarthritis 68, skin infection 69, 70, tumor 71, etc. In this study, CX3CR1-KikGR transgenic mice were generated and used to specifically track the migration of splenic CX3CR1^+^ cells from the spleen to the fibrotic liver *in vivo* (Figure 6A-L and S18C-G). We estimated that approximately 14% (∼0.57×10^4^) of photoconverted splenic CX3CR1^+^ KikRed^+^ cells (∼4.06×10^4^) migrated into the fibrotic liver (Figure S18C and S18F-G) and then contacted with hepatic CX3CR1^+^ KikGreen^+^ cells for a long time (Figure 6L and S18I). These data provide direct evidence that splenic CX3CR1^+^ cells migrated from the spleen and then accumulated into the fibrotic liver.

In summary, our study described the changes in spatial distribution and migratory behavior of hepatic CX3CR1^GFP^ cells during liver fibrosis, which were obviously reversed by splenectomy. A subset of splenic CX3CR1^+^ monocytes had characteristics of classical monocyte and migrated from the spleen and reached the fibrotic liver, causing behavioral changes of hepatic endogenous CX3CR1^GFP^ cells and exacerbating liver fibrosis. These findings shed light on the spleen-liver axis involved in the progression of liver fibrosis and suggest a unique and essential role of splenic CX3CR1^GFP^ monocytes through the spleen-liver axis in liver fibrosis precession. Therefore, targeting splenic CX3CR1^GFP^ monocytes may be beneficial in the treatment of liver fibrosis.

## Materials and methods

### Animals

In this study, we used the following animals. C57BL/6 male mice were purchased from the Hunan SJA Laboratory Animal Co., Ltd (Changsha, Hunan, China). The male CX3CR1^GFP/+^ mice (B6.129P2(Cg)-CX3CR1^tm1Litt^/J, Stock No. 005582), ROSA^mT/mG^ mice (B6.129(Cg)-Gt(ROSA)26Sor^tm4(ACTB-tdTomato,-EGFP)Luo^/J, Stock No. 007676), CX3CR1-Cre mice (B6J.B6N(Cg)- CX3CR1^tm1.1(cre)Jung^/J, Stock No. 025524) were purchased from the Jackson Laboratory. KikGR mice (B6.Cg-Gt(ROSA)26Sor^tm1(CAG-kikGR)Kgwa^, Stock No. RBRC09254) were obtained from RIKEN Bio-Resource Center Experimental Animal Division (Japan) and bred in our facility. CX3CR1-KikGR mice for experiments were generated by mating the CAG-lox-STOP-lox-kikGR ROSA26 knock-in mice with CX3CR1-Cre mice.

The male mice were housed in our animal facility for at least 1 week before the experiments and used in studies when 6-8 weeks old. All mice were maintained and bred under specific pathogen-free (SPF) conditions in the institute's animal facility. Animal experiments were in accordance with the Experimental Animal Management Ordinance of Hubei Province, P. R. China and strictly performed in compliance with protocols approved by the Animal Experimentation Ethics Committee of Huazhong University of Science and Technology.

### Experimental liver fibrosis and splenectomy

Liver fibrosis was induced by intraperitoneal injections of CCl4 (Sinopharm Chemical Reagent Co., Ltd., China) (1 mL/kg body weight; 1:3 dilution with corn oil) or corn oil (Solarbio, China) as a control twice a week for 4-6 weeks 72, 73. Animals were randomly divided into four groups: Oil-treated group with Spx or Sham, CCl4-treated group with Spx or Sham. Splenectomy was performed under completely sterile conditions as follows. A small incision was made on the left side of the abdominal cavity through the skin and peritoneum. Splenic arteries and veins were identified and ligated with 5-0 nylon sutures and the spleen was removed outside the incision. The sham-operation was performed by a laparotomy without removing the spleen. Removal of the spleen (splenectomy) or sham surgery was performed before the eighth injection of Oil or CCl4. After CCl4 administration at the sixth week, mice were euthanized and the organizations were collected for subsequent experimental studies.

### Immunofluorescence staining and Masson trichrome

For immunofluorescence staining, tissue (liver and spleen) specimens were fixed in 4 % paraformaldehyde. The sections were cut, washed in phosphate buffered saline (PBS) (Cytiva, USA), and blocked with 1% bovine serum albumin (BioFroxx, Germany) for 1 hour at room temperature. Primary antibodies were diluted in blocking solution at 1: 200 for CD68-BV421 (Clone FA-11, Cat# 137017, Biolegend), CD68-Alexa Fluor 647 (Clone FA-11, Cat# 137004, Biolegend), CD86-APC (Clone GL-1, Cat# 558703, BD Biosciences), CD206 (MMR)-APC (Clone C068C2, Cat# 141708, Biolegend), F4/80-BV421 (Clone T45-2342, Cat# 565411, BD Biosciences), F4/80-Alexa Fluor 594 (Clone BM8, Cat# 123140, Biolegend), F4/80-Alexa Fluor 647 (Clone BM8, Cat# 123122, Biolegend) and incubated at 4°C overnight, followed by washing with PBS. Nuclei were stained with DAPI (Sigma, USA). The sections were imaged by spinning disk confocal microscope UltraViewVoX (PerkinElmer, USA) with a dry 20×/0.75 NA Objective (Olympus, Japan). The data were quantified by ImageJ software (NIH, USA). Masson's trichrome staining was used to evaluate and examine the histopathologic changes in liver structure including pathological collagen accumulation. The sections were imaged with a Nikon Ni-E (Nikon, Minato, Japan) with a dry 10×/0.45 NA Objective. For each specimen, 5 fields per tissue section were randomly chosen and quantified by Image J software (National Institutes of Health, USA).

### Non-parenchymal liver cell preparation

After the mice were anesthetized, the liver was perfused via the portal vein for 15 minutes with PBS at 37 °C until the liver was completely discolored. The liver was dissected into pieces and digested using 0.5 mg/ml collagenase IV (Worthington, USA) and 0.2 mg/ml DNAase I (Sigma-Aldrich, USA) for 30 minutes at 37°C as described previously. The digested liver extracts were filtered through a 70 μm cell strainer and centrifuged at 500 × g for 5 minutes. Part of the sorted cells were labeled by antibodies to identify the CD45^+^ population. The remaining cells were resuspended in 20 mL 36.5% Percoll (Cytiva, USA) and centrifuged at 700 × g for 20 minutes at room temperature. Non-parenchymal liver cells were collected and resuspended in 3 mL red blood cell lysis solution (Biosharp, China). After incubation for 3 minutes at room temperature, cells were washed twice with RPMI 1640 (Cytiva, USA) containing 2% FBS (Gibco, USA).

### Flow cytometry

Flow cytometry analysis for monocyte/macrophages in organs was performed according to the manufacturer's instructions. The single-cell suspension was passed through a 70 μm cell strainer and centrifuged at 3,000 rpm for 5 minutes at 4℃. The cell viability was assessed using the fixable viability dye eFluor506 (Cat# 65-0866-18, ThermoFisher, USA) and then the single cells were incubated with the following antibodies. CD4-BV421 (Clone GK1.5, Cat# 562891, BD Bioscience), CD8-Alexa Fluor 647 (Clone 53-6.7, Cat# 100724, Biolegend), B220-APC-Cy7 (Clone RA3-6B2, Cat# 103223, Biolegend), CD19-PE-Cy7 (Clone 1D3, Cat# 552854, BD Bioscience), Ly6C-PE (Clone HK1.4, Cat# 128008, Biolegend), CD11b-PE-Cy7 (Clone M1/70, Cat# 552850, BD Biosciences), Ly6G-APC/Cy7 (Clone 1A8, Cat# 127624, Biolegend), CD45-Alexa Fluor 700 (Clone 30-F11, Cat# 103128, Biolegend), CD68-BV421 (Clone FA-11, Cat# 137017, Biolegend), CD68-Alexa Fluor 647 (Clone FA-11, Cat# 137004, Biolegend), CD11c-BV421 (Clone N418, Cat# 565452, BD Biosciences), CD11c-APC-Cy7 (Clone HL3, Cat# 561241, BD Bioscience), F4/80-BV421 (Clone T45-2342, Cat# 565411, BD Biosciences), CD115-APC (Clone AFS98, Cat# 17-1152-82, Invitrogen), MHC-Ⅱ (I-A/I-E)-PE (Clone M5/114.15.2, Cat# 107608, Biolegend) were used for flow cytometry (FCM). Cells were analyzed using a CytoFLEX flow cytometer (Beckman Coulter, Brea, CA, USA). Flow cytometry data were analyzed using FlowJo software (FlowJo, LLC, Ashland, USA).

### Intravital imaging

The mice were anesthetized by i.p. injection of a mixture of 10 mg/kg xylazine and 100 mg/kg ketamine hydrochloride (Sigma, St. Louis, Missouri, USA). 5 μg of Alexa Fluor 647 anti-mouse CD31 antibody (Clone:390, Cat# 102416, Bio Legend) was diluted in sterile saline (total volume of 100 μl) and injected into mice via the lateral tail vein before imaging. Next, the abdominal cavity was depilated, and then skin outside the spleen or liver was cut to directly expose the spleen or liver as previous research has reported 31. Mice were anesthetized with 0.5-1.0% isoflurane in oxygen flow at 0.6 L/min controlled by a small animal anesthesia machine (RWD, China) and placed within a custom-designed imaging box. Intravital imaging was performed by using a spinning disk confocal microscope UltraViewVoX (PerkinElmer, USA). GFP and RFP fluorescent signals were separately excited by the 488 nm or 647 nm laser. All fluorescence images were observed using a 20×/0.75 NA objective (Olympus, Japan) and were acquired using Volocity (Version 6.1.1, PerkinElmer) software. Image data were processed with Image J software (National Institutes of Health, USA), Imaris (Version 7.4.2, Bitplane) software and MATLAB (MathWorks, USA). When beginning the imaging procedures, about 20-minute imaging sequences were monitored using a 20×/0.75 NA objective. By tracking individual ending of the process of interacting CX3CR1^GFP^ cell and the center of mobile CX3CR1^GFP^ cell from their starting position to their final position over time in two dimensions, the migratory behavior of mobile CX3CR1^GFP^ cells and interacting CX3CR1^GFP^ cells processes can be qualitatively analyzed. The migratory trajectory of mobile CX3CR1^GFP^ cells was tracked via automatic spot analysis in Imaris software, whereas the trajectory of interacting CX3CR1^GFP^ cells processes was tracked manually. The trajectory, velocity, confinement ratio, and arrest coefficient were acquired in MATLAB.

### Purification of splenic monocytes/macrophages for adoptive transfer

To collect cells from the spleen, the entire spleen was aseptically removed and minced through a nylon mesh in cold PBS. The single-cell suspensions in PBS were filtered through a 70 μm strainer to remove tissue debris. Filtered cells were centrifuged at 3,000 rpm for 5 minutes at 4℃. Splenocytes were isolated from ROSA^mT/mG^ fibrotic donors and splenic CX3CR1^GFP^ cells were isolated from CX3CR1^(GFP/+)^ fibrotic donors. The splenic cells were washed with PBS and centrifuged for 5 minutes at 3,000 rpm. To purify splenic CX3CR1^GFP^ cells, cells were first enriched using EasySep™ Mouse CD11b Positive Selection Kit II (STEMCELL, Cat# 18970A, Canada) according to the manufacturer's protocol, then flow-sorted (FACSAria III, BD Biosciences) for GFP expression by selecting leucocytes according to their size and excluding doublets (GFP expression within an FSC/SSC gate appropriate for monocytes). Following these procedures, monocytes were washed twice in PBS by centrifugation at 3,000 rpm for 5 minutes at 4℃ and were resuspended in PBS. The splenic classical monocytes (CD11b^+^ CD115^+^ CX3CR1^low^ Ly6C^high^) and non-classical monocytes (CD11b^+^ CD115^+^ CX3CR1^high^ Ly6C^low^) were enriched via the same sorting strategy, which led to the purity of sorted cells was more than 90%. The collected splenic cells (1×10^7^ cells/mouse), CMTPX (CellTracker Red CMTPX, Eugene, OR) labeled CD11b^+^ CX3CR1^+^ cells (5×10^5^ cells/mouse), CMTPX labeled CD11b^+^ CD115^+^ CX3CR1^low^ Ly6C^high^ monocytes (1×10^5^ cells/mouse) and CD11b^+^ CD115^+^ CX3CR1^high^ Ly6C^low^ monocytes (3.5×10^4^ cells/mouse) suspended in 200 μl PBS were adoptively transferred i.v. into either male splenectomized CX3CR1^(GFP/+)^ or C57BL/6 mice prior to CCl4 injection. 24 h or 14 d after the 8th CCl4 injection, the mice were euthanized and the organizations were collected for subsequent experimental studies.

### Quantitative PCR

The liver tissue (caudate lobe) was lysed with TRIzol reagent (Invitrogen, Carlsbad, CA, USA) and total RNA of tissue was extracted according to the manufacturer's protocol. The quantitative PCR was performed according to the manufacturer's instructions. Reverse transcription was performed with equal amounts of RNA using the PrimeScript RT Reagent Kit with gDNA Eraser (Takara, Dalian, China) and PCR was performed using the StepOnePlus™ Real-Time PCR System (Applied Biosystems). Quantitative targeted gene expression data were normalized to the expression levels of beta-actin and were analyzed by the relative quantification (ΔΔCt) method. Primer sequences were listed in Supplementary Table 1.

### Photoconversion surgery

Photoconversion of the spleen of CX3CR1-KikGR mice was performed as described previously 71. Briefly, CX3CR1-KikGR mice were anesthetized, the abdominal skin was incised and the spleen was exposed to violet light (three sides × 3 minutes, TOP UV Flashlight equipment with a 405 nm laser) (TaoYuan, China). Two pieces of sterile aluminum foil were arranged on either side of the spleen, shielding the skin and abdominal cavity. After photoconversion, the spleen was replaced, and the abdominal cavity and the skin were sutured. The unconverted group (no surgery), 0-h group (killed before photoconversion) and photoconversion group (performed photoconversion) were designed in each experiment to precisely assess photoconversion efficiency as previous research has described.

### Statistical analysis

All statistical analyses were performed with GraphPad Prism 8 (GraphPad Software, USA). For comparisons of two groups, unpaired Student's* t*-test or Mann-Whitney test was used. For comparisons of three or more groups, one-way ANOVA test followed by Tukey's multiple comparisons post-test, Brown-Forsythe and Welch ANOVA tests or the Kruskal-Wallis test followed by Dunn's multiple comparisons post-test was used. Experimental data were presented as mean ± SEM. Differences between or among groups are denoted as ns for not significant, * for *P* < 0.05, ** for *P* < 0.01, *** for *P* < 0.001, and **** for *P* < 0.0001.

## Supplementary Material

Supplementary figures, table, movie legends.

Supplementary movie 1.

Supplementary movie 2.

Supplementary movie 3.

Supplementary movie 4.

Supplementary movie 5.

Supplementary movie 6.

Supplementary movie 7.

Supplementary movie 8.

Supplementary movie 9.

Supplementary movie 10.

Supplementary movie 11.

## Abbreviations

CX3CR1: CX3C chemokine receptor1
CX3CL1: Fractalkine (C-X3-C motif) ligand 1
CCl4: Carbon tetrachloride
CV: central vein
PT: portal triad
Mon/Mφs: monocyte and macrophages
KikGR: Kikume Green-Red
CTLs: Cytotoxic T Lymphocytes
AT: adoptive transfusion
Mon: monocyte
α-SMA: alpha-smooth muscle actin
COL1A1: Collagen type I alpha 1
Spx: splenectomy
iNOS: Inducible nitric oxide synthase
GFP: green fluorescent protein
RFP: red fluorescent protein
DCs: dendritic cells
IL-1β: interleukin-1beta
TNF-a: Tumor necrosis factor-alpha
HSCs: hepatic stellate cells
FBS: fetal bovine serum
PBS: phosphate buffered saline
SPF: specific pathogen-free
