# Supplementary material for: Intravital imaging of splenic classical monocytes modifying the hepatic CX3CR1+ cells motility to exacerbate liver fibrosis via spleen-liver axis
Source: Theranostics. 2024 Mar 3;14(5):2210–31. doi: 10.7150/thno.87791 (PMC10945343; doi:10.7150/thno.87791)
Supplement: Supplementary file 1 — Supplementary figures, table, movie legends. [file thnov14p2210s1.pdf]

# Intravital imaging of splenic classical monocytes modifying the hepatic $CX3CR1^+$ cells motility to exacerbate liver fibrosis via spleen-liver axis

Chenlu Han[1], Yujie Zhai[1], Yuke Wang[1], Xuwen Peng[1], Xian Zhang[1], Bolei Dai[1], Yuehong Leng[1], Zhihong Zhang[1,2]* and Shuhong Qi[1]*

[1]Britton Chance Center and MoE Key Laboratory for Biomedical Photonics, Wuhan National Laboratory for Optoelectronics-Huazhong University of Science and Technology, Wuhan, Hubei 430074, China

[2]State key laboratory of digital medical engineering, School of Biomedical Engineering, Hainan University, Haikou, Hainan 570228, China

* Correspondence: Zhihong Zhang, czyzzh@mail.hust.edu.cn;
Shuhong Qi, qishuhong@hust.edu.cn;

Address: Room G304, Britton Chance Center for Biomedical Photonics, Wuhan National Laboratory for Optoelectronics-Huazhong University of Science and Technology, Wuhan, Hubei 430074, China
Fax: +86-27-87792034; Tel: +86-27-87792033;

Zhihong Zhang (czyzzh@mail.hust.edu.cn) and Shuhong Qi (qishuhong@hust.edu.cn) are the corresponding authors for communication with the Editorial and Production offices.

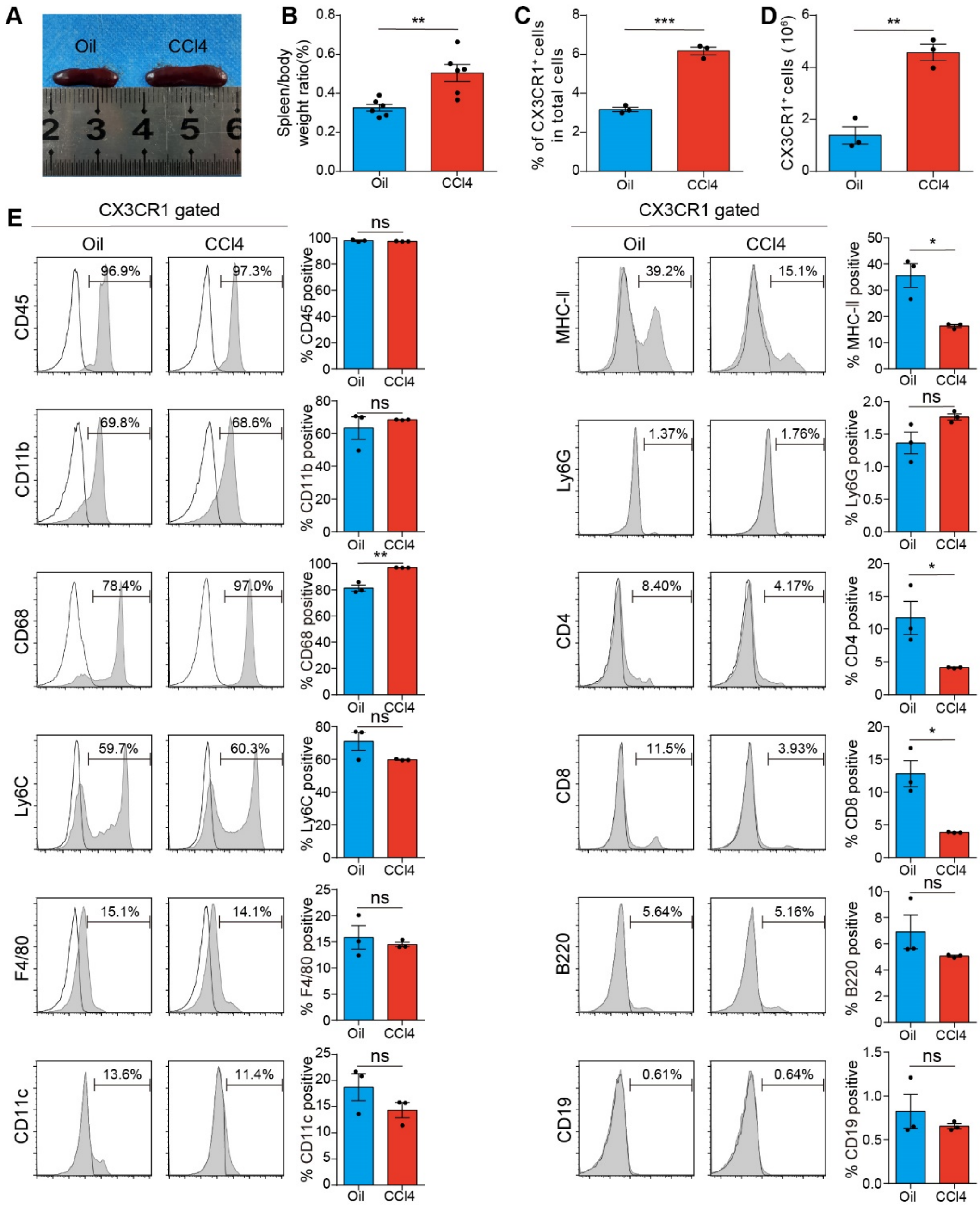


**Figure S1. Flow cytometry analysis of splenic CX3CR1$^+$ cells and their phenotypic characteristics in Oil/CCl4-treated mice.** (**A**) Spleen in the Oil and CCl4-treated mice. (**B**) Spleen/body weight ratio was calculated (n = 6 mice per group). (**C**) The percentage of splenic CX3CR1$^+$ cells in Oil-treated mice or CCl4-treated mice (n = 3 mice per group). (**D**) The number of splenic CX3CR1$^+$ cells in Oil-treated mice or CCl4-treated mice (n = 3 mice per group). (**E**) The phenotypic characteristics of splenic CX3CR1$^+$ cells in Oil-treated mice or CCl4-treated mice (n = 3 mice per group). Data are presented as the mean ± SEM.

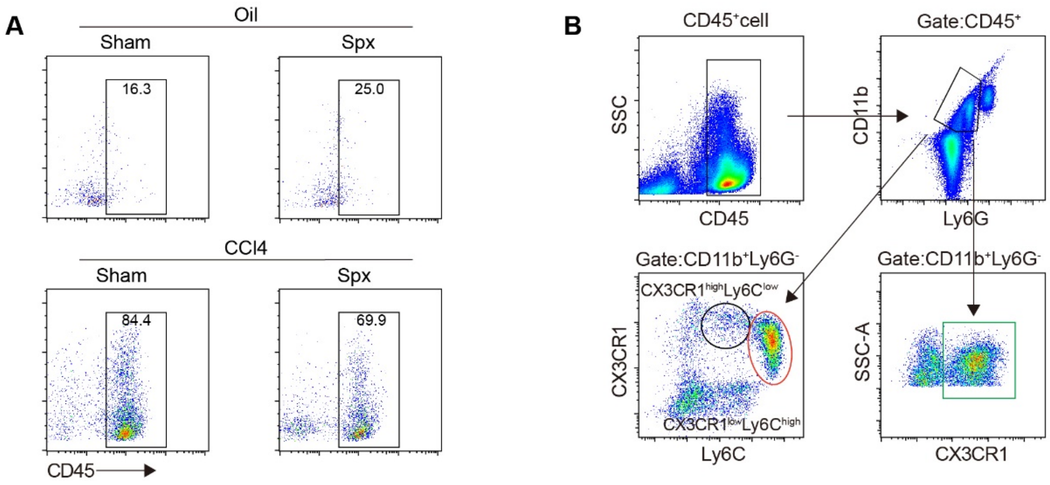


**Figure S2. Flow cytometry analysis of hepatic CX3CR1$^{+}$ cells and their subsets in Oil/CCl4-treated mice with or without splenectomy.** (**A**) Gating strategy of hepatic CD45$^{+}$ cells. (**B**) Gating strategy of hepatic CX3CR1$^{+}$ cells and their subsets.

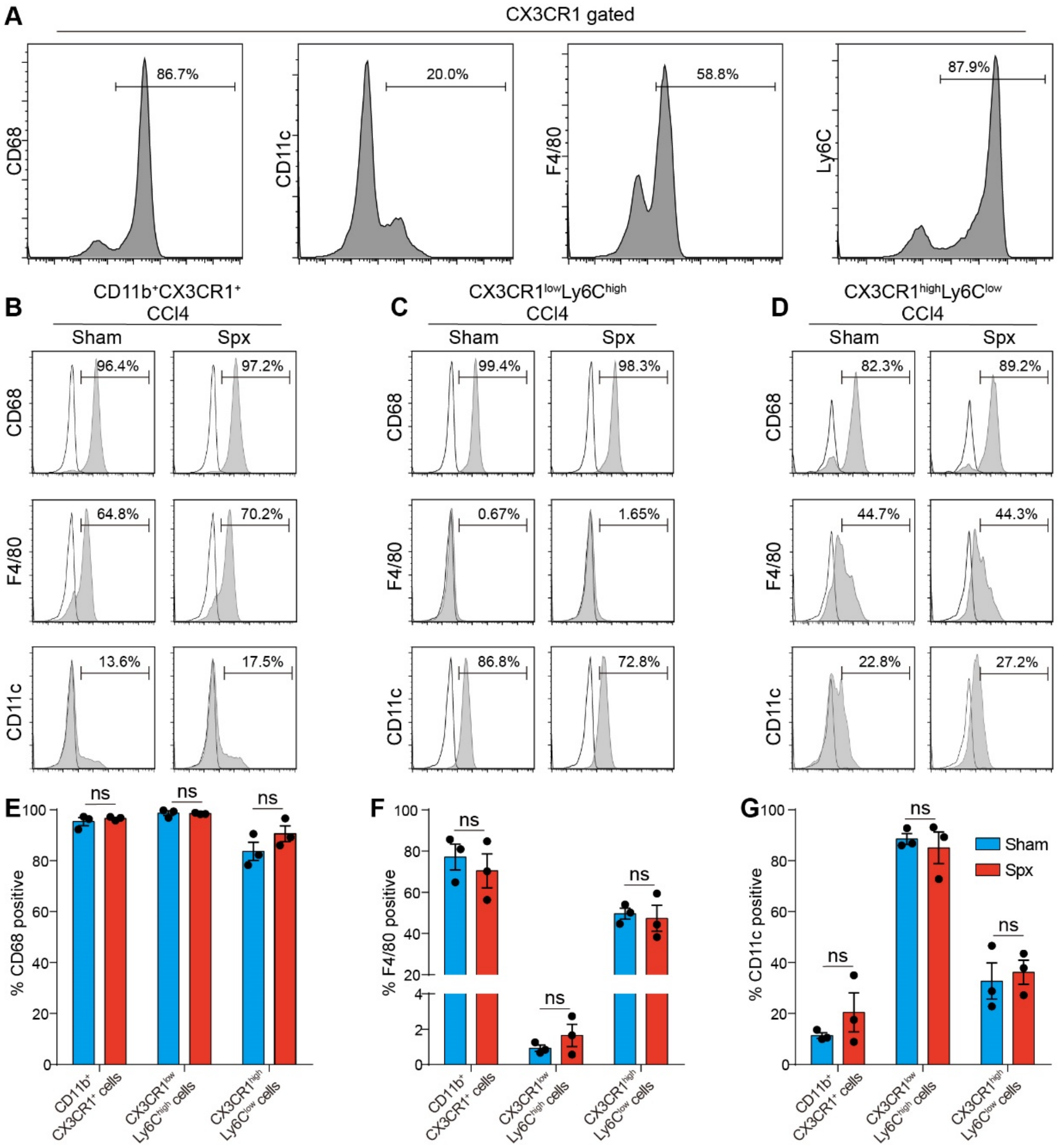


**Figure S3. Phenotypic characteristics of CX3CR1$^+$ cells and their subsets in the livers of CCl4-treated mice with or without splenectomy.** (**A**) Phenotypic analysis of hepatic CX3CR1$^+$ cells from CCl4-treated mice by flow cytometry. (**B-D**) The hepatic CX3CR1$^+$ cells and their subsets have distinct phenotypic profiles. (**E-G**) Phenotypic analysis of hepatic CD11b$^+$ CX3CR1$^+$ cells and their subsets from fibrotic mice at one week after splenectomy by flow cytometry (n = 3 mice per group). Data are presented as the mean ± SEM.

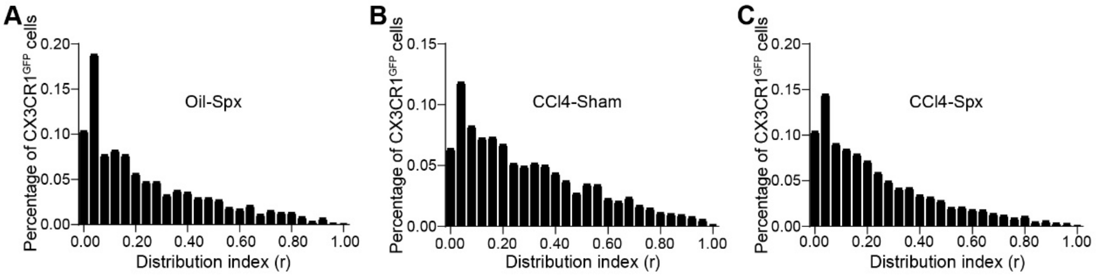


**Figure S4. The distribution of CX3CR1$^{GFP}$ cells in each distribution index (r) from different groups.** (**A**) The distribution of CX3CR1$^{GFP}$ cells in each distribution index (r) from Oil-treated group with Spx; the bin value is 0.04. (**B**) The distribution of CX3CR1$^{GFP}$ cells in each distribution index (r) from CCl4-treated group with Sham; the bin value is 0.04. (**C**) The distribution of CX3CR1$^{GFP}$ cells in each distribution index (r) from CCl4-treated group with Spx; the bin value is 0.04.

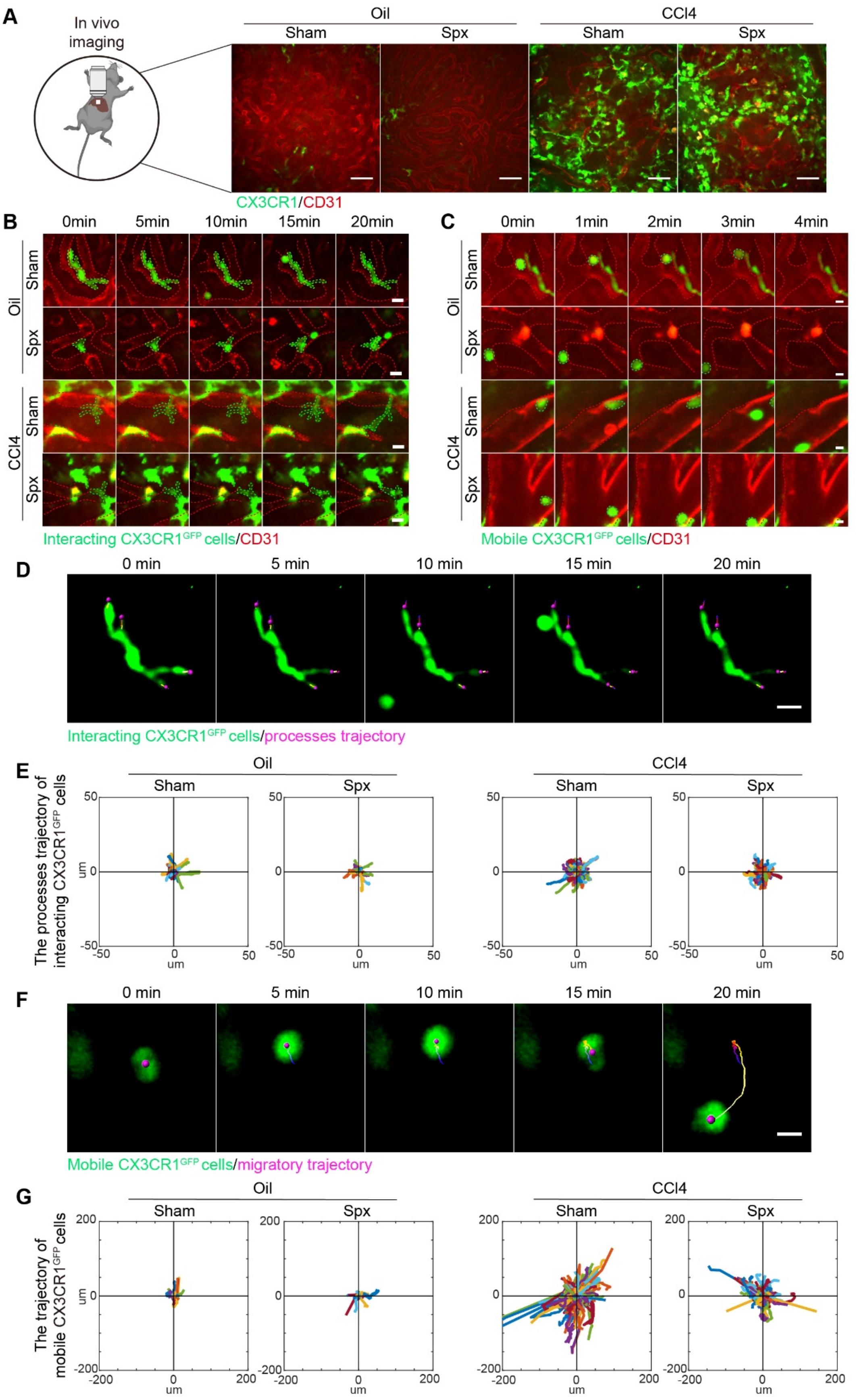


**Figure S5. Intravital imaging of hepatic CX3CR1$^{GFP}$ cell subtypes in the Oil/CCl4-treated mice with or without splenectomy.** (**A**) Representative fluorescence images of CX3CR1$^{GFP}$ cells

in the liver of Oil/CCl4-treated mice with or without splenectomy. Green: CX3CR1$^{GFP}$ cells; Red: AF647 anti-CD31 labeled hepatic vessels. Scale bar, 50 μm. (**B**) The interacting CX3CR1$^{GFP}$ cells with motile processes cause distinct shape changes during 20 minutes. Green: CX3CR1$^{GFP}$ cells; Red: AF647 anti-CD31 labeled hepatic vessels. Scale bar, 10 μm. (**C**) The rapid movement of mobile CX3CR1$^{GFP}$ cells in the hepatic vessels. Green: CX3CR1$^{GFP}$ cells; Red: AF647 anti-CD31 labeled hepatic vessels. Scale bar, 2 μm. (**D**) Representative images showing the processes trajectory of interacting CX3CR1$^{GFP}$ cell over a 20-minute period. Scale bar, 10 μm. (**E**) The processes trajectory of interacting CX3CR1$^{GFP}$ cells in the liver parenchyma. (**F**) Representative images showing the migratory trajectory of mobile CX3CR1$^{GFP}$ cell over a 20-minute period. Scale bar, 5 μm. (**G**) The trajectory of mobile CX3CR1$^{GFP}$ cells in the hepatic vessels.

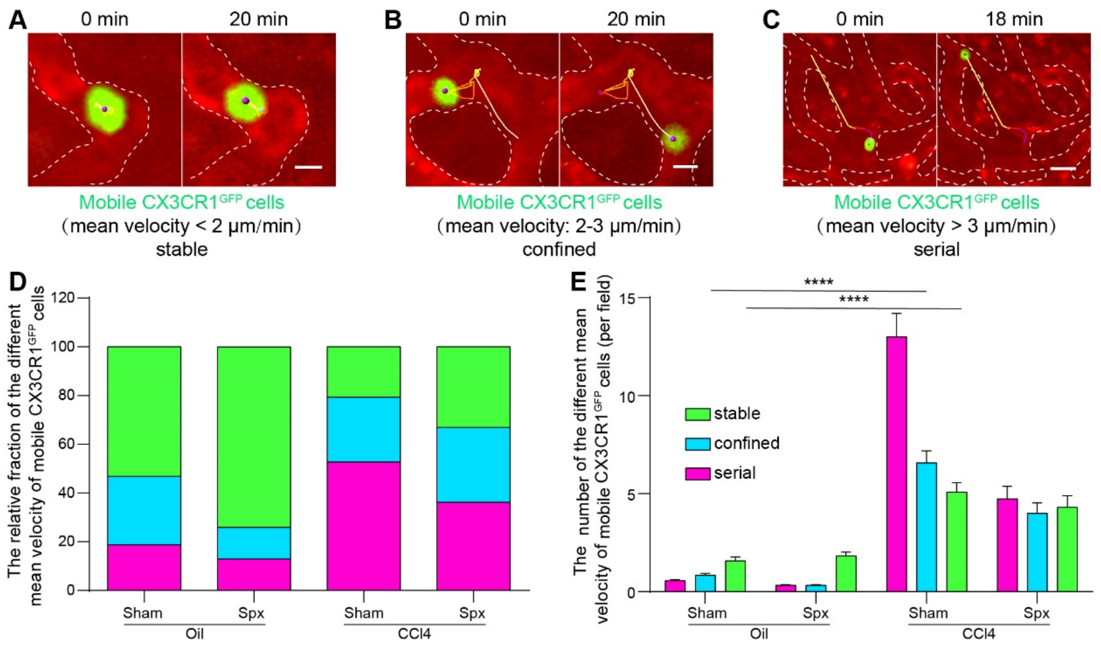


**Figure S6. The different mobile type of hepatic CX3CR1$^{GFP}$ cell in the Oil/CCl4-treated mice with or without splenectomy.** (**A**) Representative images showing the migratory trajectory of mobile CX3CR1$^{GFP}$ cell with mean velocity less than 2 µm/min ("stable" type) over a 20-minute period. Green: CX3CR1$^{GFP}$ cells; Red: AF647 anti-CD31 labeled hepatic vessels. White dotted line shows the vessels. Scale bar, 5 µm. (**B**) Representative images showing the migratory trajectory of mobile CX3CR1$^{GFP}$ cell with mean velocity between 2-3 µm/min ("confined" type) over a 20-minute period. Green: CX3CR1$^{GFP}$ cells; Red: AF647 anti-CD31 labeled hepatic vessels. White dotted line shows the vessels. Scale bar, 5 µm. (**C**) Representative images showing the migratory trajectory of mobile CX3CR1$^{GFP}$ cell with mean velocity more than 3 µm/min ("serial" type) over a 20-minute period. Green: CX3CR1$^{GFP}$ cells; Red: AF647 anti-CD31 labeled hepatic vessels. White dotted line shows the vessels. Scale bar, 15 µm. (**D**) The relative fraction of the different mobile type of CX3CR1$^{GFP}$ cells in the liver of Oil/CCl4-treated mice with or without splenectomy (n = 22-28 fields, from 3 mice per group). (**E**) The number of the different mobile type of CX3CR1$^{GFP}$ (per field) in the liver of Oil/CCl4-treated mice with or without splenectomy (n = 22-28 fields, from 3 mice per group).

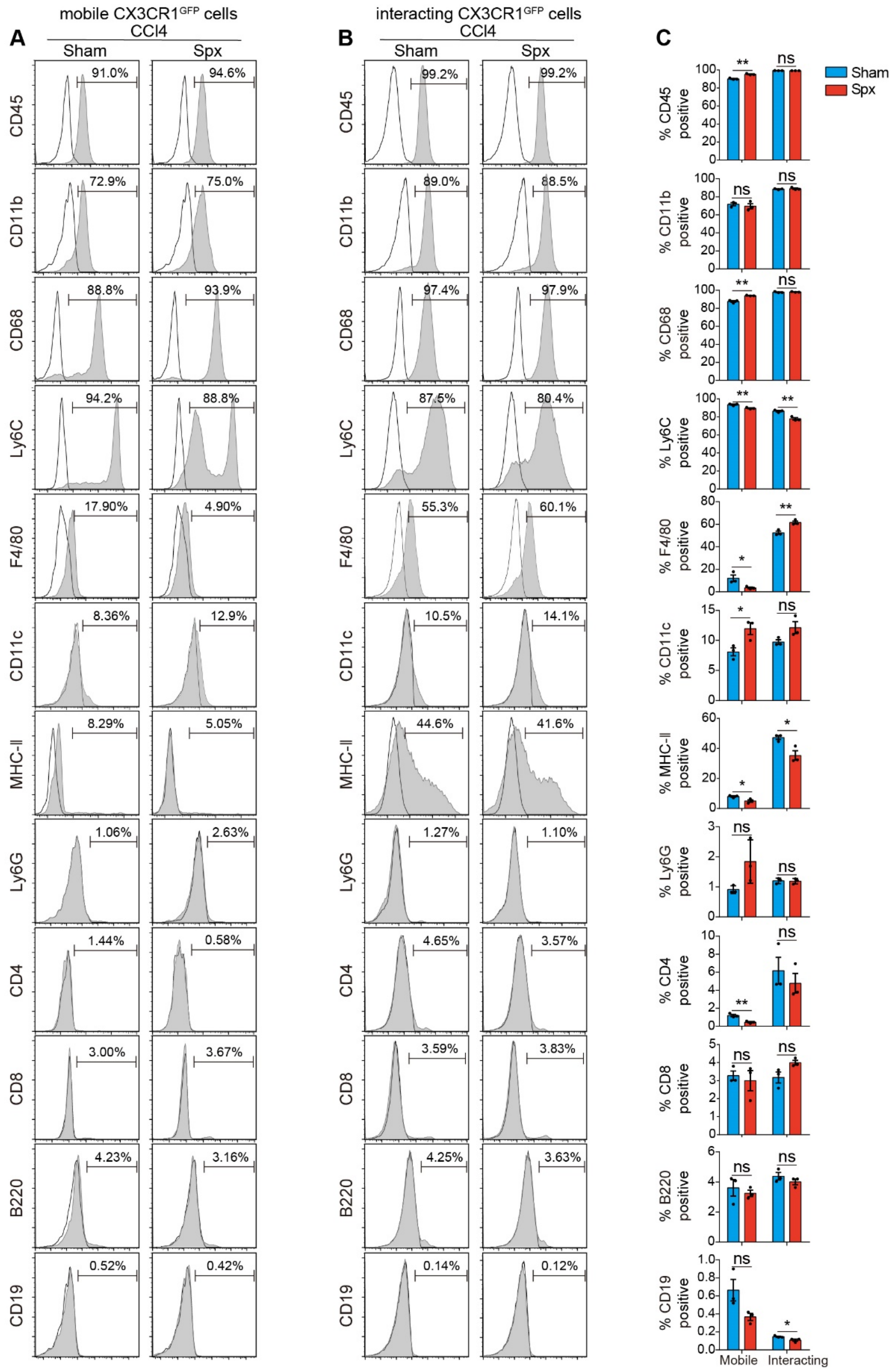


**Figure S7. Phenotypic characteristics of mobile CX3CR1$^{GFP}$ cells and interacting CX3CR1$^{GFP}$ cells in the livers of CCl4-treated mice with or without splenectomy.** (**A-B**) The surface marker profile of hepatic mobile CX3CR1$^{GFP}$ cells and interacting CX3CR1$^{GFP}$ cells. (**C**) Phenotypic analysis of hepatic mobile CX3CR1$^{GFP}$ cells and interacting CX3CR1$^{GFP}$ cells from fibrotic mice at

24 h after splenectomy by flow cytometry (n = 3 mice per group). Data are presented as the mean ± SEM.

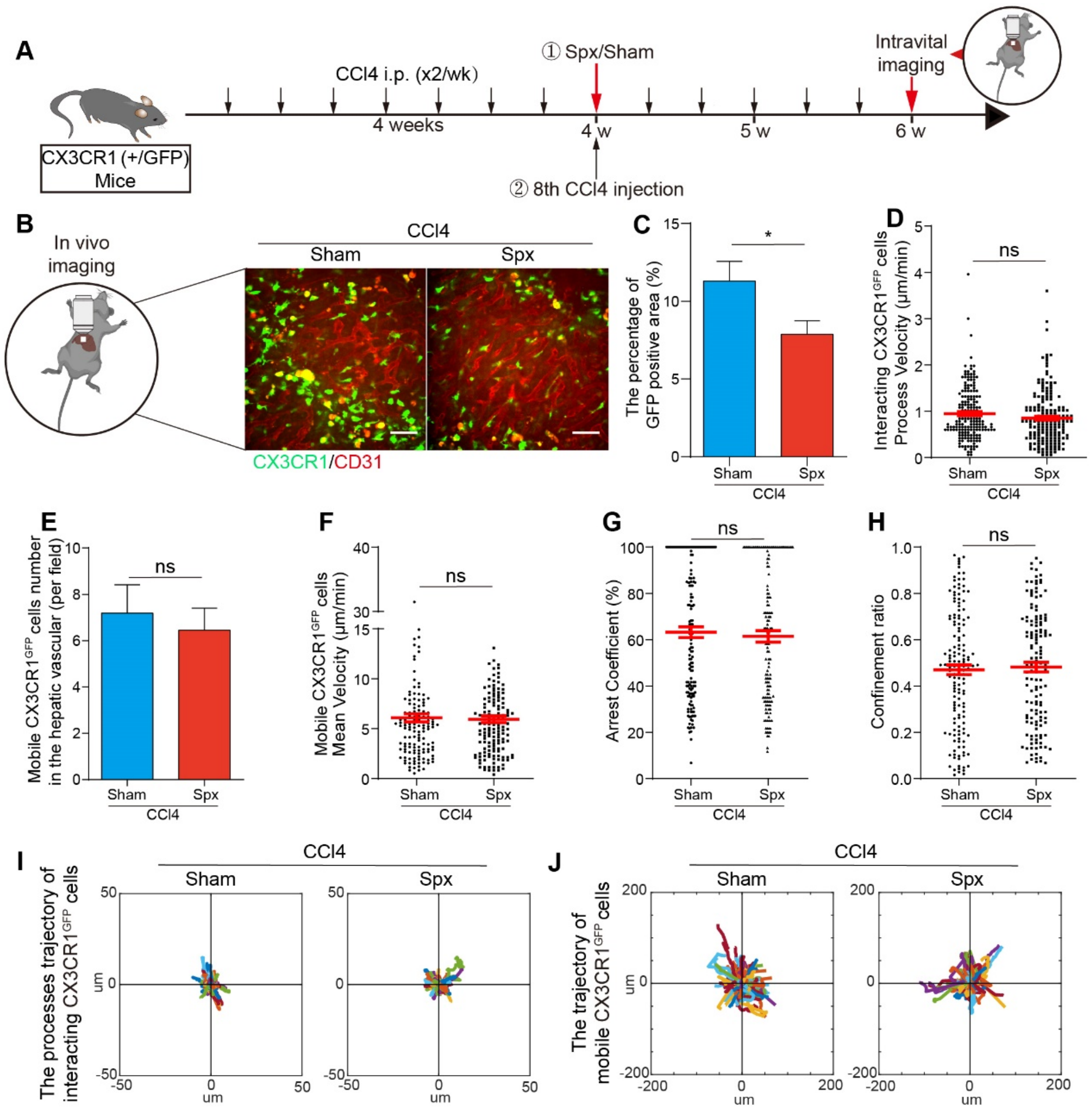


**Figure S8. Intravital imaging of CX3CR1$^{GFP}$ cells in the livers of CCl4-treated mice with or without splenectomy at 14 days after splenectomy.** (**A**) Schedules of generation of liver fibrosis model, the splenectomy and intravital imaging. (**B**) Representative fluorescence images of the CX3CR1$^{GFP}$ cells in the livers of CCl4-treated mice with or without splenectomy. Green: CX3CR1$^{GFP}$ cells; Red: AF647 anti-CD31 labeled hepatic vessels. Scale bar, 50 µm. (**C**) The positive areas of GFP were quantified using Image J software (from 3 mice per group). (**D**) The velocity of interacting CX3CR1$^{GFP}$ cells processes in the liver parenchyma. Each dot represents a single cell process, and the red bars indicate mean values (3 mice per group). (**E**) The cell number of mobile CX3CR1$^{GFP}$ cells in the hepatic vessels of CCl4-treated mice with or without splenectomy (n = 21-22 fields, from 3 mice per group). (**F-H**) Scatter plots of mean velocity (**F**), arrest coefficient (**G**), and confinement ratio (**H**) of mobile CX3CR1$^{GFP}$ cells in the hepatic vessels of CCl4-treated mice with or without splenectomy (n = 21-22 fields, from 3 mice per group). Each dot represents a

single cell, and the red bars indicate mean values. Data are presented as mean ± SEM. (**I**) The processes trajectory of interacting CX3CR1$^{GFP}$ cells in the liver parenchyma. (**J**) The trajectory of mobile CX3CR1$^{GFP}$ cells in the hepatic vessels.

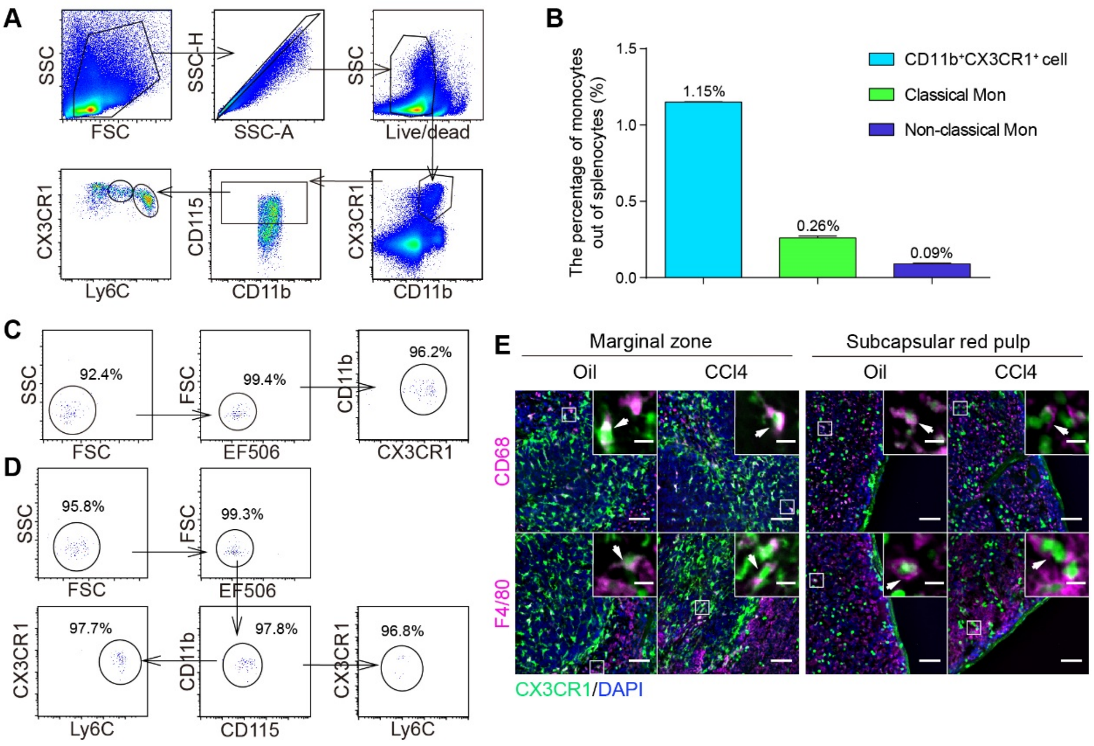


**Figure S9. Flow cytometry analysis and immunofluorescence staining to identify splenic CX3CR1$^+$ cells and their subsets in the spleen of Oil/CCl4-treated mice.** (**A**) Gating strategy of splenic classical monocytes and non-classical monocytes. (**B**) The percentage of CD11b$^+$ CX3CR1$^+$ cells, classical monocytes and non-classical monocytes out of splenocytes in the fibrotic spleen (n = 3 mice per group). (**C-D**) The percentage of sorted splenic CD11b$^+$ CX3CR1$^+$ cells (**C**), CD11b$^+$ CD115$^+$ CX3CR1$^{low}$ Ly6C$^{high}$ cells and CD11b$^+$ CD115$^+$ CX3CR1$^{high}$ Ly6C$^{low}$ cells (**D**). (**E**) The cell surface receptor profile of CX3CR1$^{GFP}$ cells in the marginal zone and subcapsular red pulp of the fibrotic spleen was examined by immunofluorescence staining. Green: CX3CR1$^{GFP}$ cells; Blue: DAPI; Magenta: CD68/ F4/80. Scale bar (large image), 50 μm. Scale bar (small image), 10 μm. White arrows indicate CX3CR1$^{GFP}$ cells.

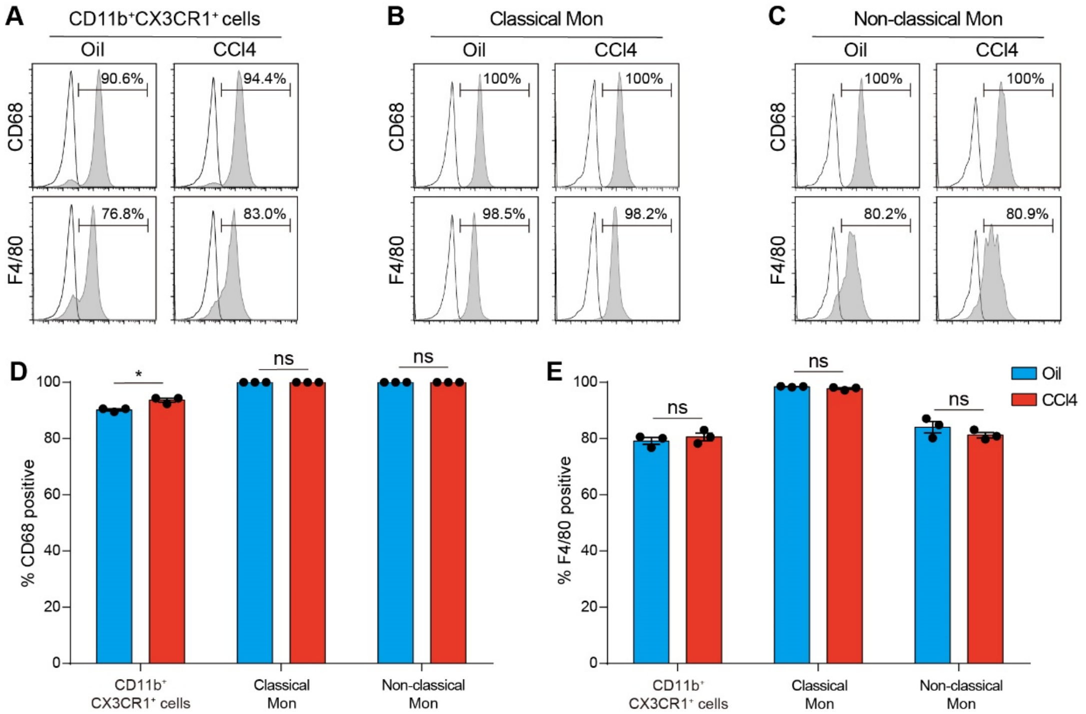


**Figure S10. Phenotypic characteristics of splenic CX3CR1$^+$ cells and their subsets from Oil/CCl4-treated mice by flow cytometry.** (**A-C**) The splenic CX3CR1$^+$ cells and their subsets have distinct phenotypic profiles. (**D-E**) Phenotypic analysis of CD11b$^+$ CX3CR1$^+$ cells and their subsets in the spleen of Oil and CCl4-treated mice by flow cytometry (n = 3 mice per group). Data are presented as the mean ± SEM.

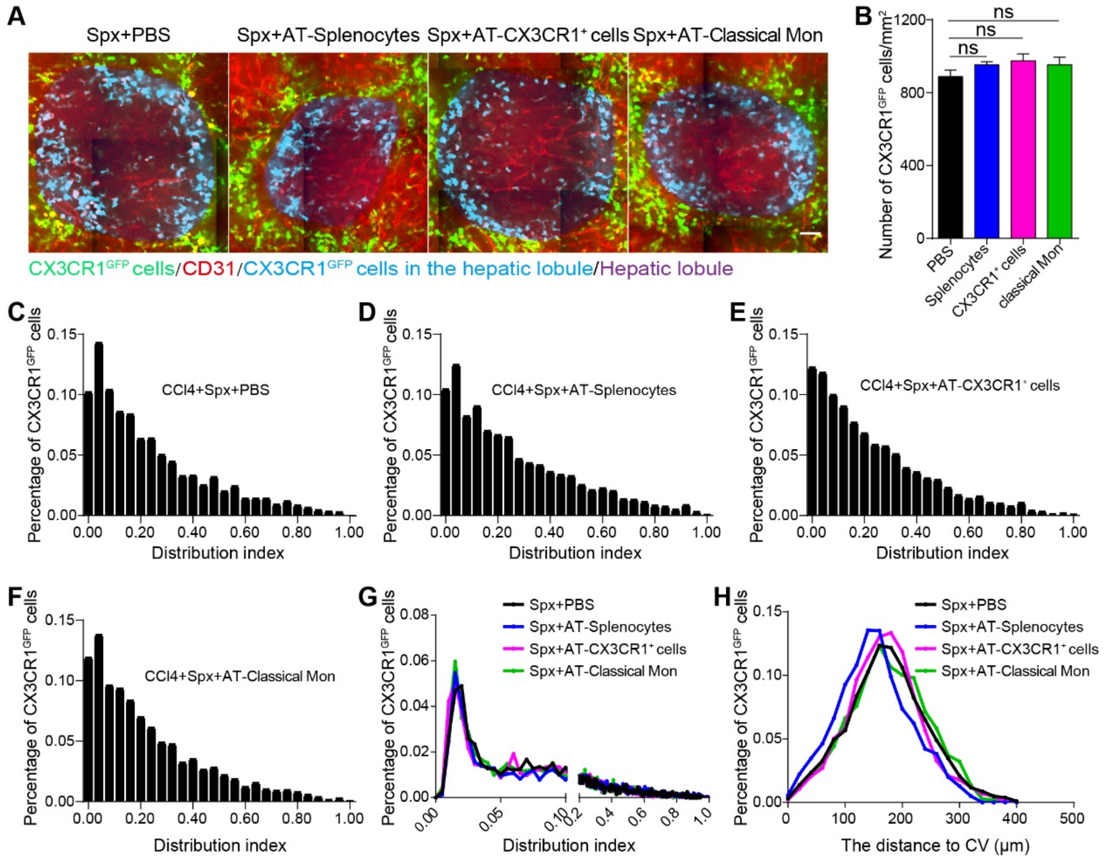


**Figure S11. Intravital imaging of the spatial distribution of CX3CR1$^{GFP}$ cells in the fibrotic liver after adoptively transferring splenic cells.** (**A**) Intravital imaging of CX3CR1$^{GFP}$ cells distribution in the hepatic lobule. Green: CX3CR1$^{GFP}$ cells; Red: AF647 anti-CD31 labeled hepatic vessels; Blue: CX3CR1$^{GFP}$ cells in the hepatic lobule; Magenta: Hepatic lobule. Scale bar, 50 µm. (**B**) The density of CX3CR1$^{GFP}$ cells in one hepatic lobule (n = 10-21, from 3 mice per group). Data are presented as mean ± SEM. (**C-F**) The distribution of CX3CR1$^{GFP}$ cells in each distribution index from different groups; the bin value is 0.04. (**G**) The distribution curves of CX3CR1$^{GFP}$ cells in each distribution index from different groups; the bin value is 0.005. (**H**) The distance of CX3CR1$^{GFP}$ cells to the hepatic central vein (CV) in the hepatic lobules.

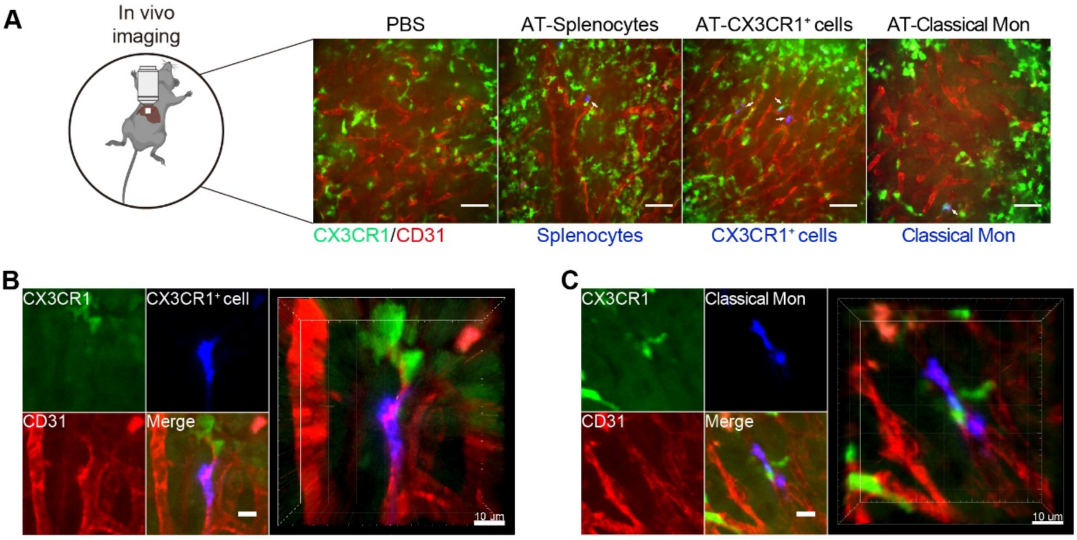


**Figure S12. Imaging of the migration behavior of endogenous CX3CR1$^{GFP}$ cells and adoptive splenic cells in the fibrotic liver.** (**A**) Intravital imaging of adoptive splenic cells in the liver. Green: CX3CR1$^{GFP}$ cells; Red: AF647 anti-CD31 labeled hepatic vessels; Blue: Splenocytes. Scale bar, 50 μm. White arrows indicate the adoptive splenic cells. (**B**) Immunofluorescence imaging of adoptive splenic CX3CR1$^{+}$ cells in the liver. Green: CX3CR1$^{GFP}$ cells; Red: AF647 anti-CD31 labeled hepatic vessels; Blue: Splenic CX3CR1$^{+}$ cells. Scale bar, 10 μm. (**C**) Immunofluorescence imaging of adoptive splenic classical monocytes in the liver. Green: CX3CR1$^{GFP}$ cells; Red: AF647 anti-CD31 labeled hepatic vessels; Blue: Splenic classical monocytes. Scale bar, 10 μm.

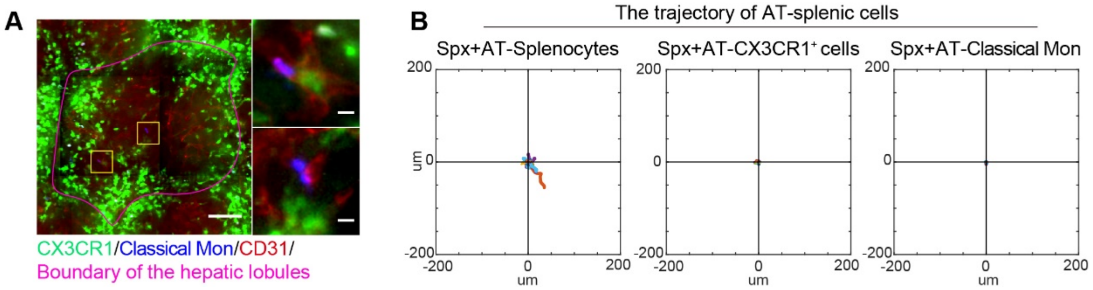


**Figure S13. Intravital imaging revealed the localization and migration behavior of adoptive splenic cells in the fibrotic liver after adoptively transferring splenic cells.** (**A**) The distribution of adoptive splenic classical monocytes in the hepatic lobules. Green: CX3CR1$^{GFP}$ cells; Blue: Classical monocytes; Red: AF647 anti-CD31 labeled hepatic vessels; Magenta: Boundary of the hepatic lobules. Scale bar, 100 μm. High-magnification views of the adoptive splenic classical monocytes in the liver are shown on the right. Scale bar, 10 μm. (**B**) The trajectory of adoptive splenic cells in different groups.

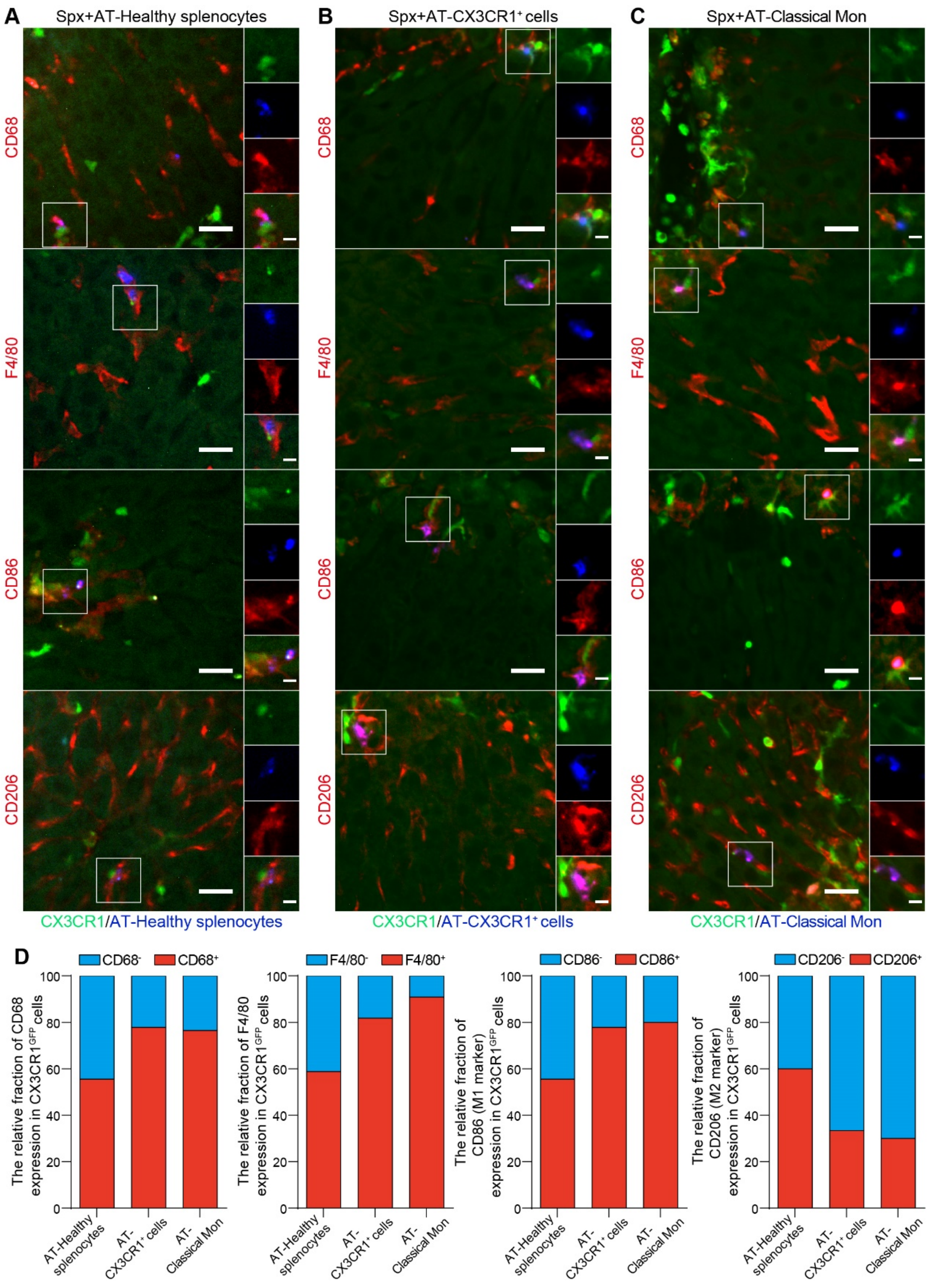


**Figure S14. The phenotypic characteristics of neighboring endogenous CX3CR1$^{GFP}$ cells contacted with adoptive splenic cells in the fibrotic liver.** (**A**) Phenotypic characteristics of neighboring endogenous CX3CR1$^{GFP}$ cells contacted with adoptive splenocytes from healthy mice. Scale bar (large image), 25 μm. Scale bar (small image), 8 μm. (**B**) Phenotypic characteristics of neighboring endogenous CX3CR1$^{GFP}$ cells contacted with adoptive splenic CX3CR1$^{+}$ cells from

fibrotic mice. Scale bar (large image), 25 μm. Scale bar (small image), 8 μm. (**C**) Phenotypic characteristics of neighboring endogenous $CX3CR1^{GFP}$ cells contacted with adoptive splenic classical monocytes from fibrotic mice. Scale bar (large image), 25 μm. Scale bar (small image), 8 μm. (**D**) Phenotypic analysis of neighboring endogenous $CX3CR1^{GFP}$ cells contacted with adoptive splenic cells (The proportion of CD68 expression in neighboring endogenous $CX3CR1^{GFP}$ cells: n = 9-18 cells, from 3 mice per group; The proportion of F4/80 expression in neighboring endogenous $CX3CR1^{GFP}$ cells: n = 17-33 cells, from 3 mice per group; The proportion of CD86 expression in neighboring endogenous $CX3CR1^{GFP}$ cells: n = 9-10 cells, from 3 mice per group; The proportion of CD206 expression in neighboring endogenous $CX3CR1^{GFP}$ cells: n = 9-10 cells, from 3 mice per group).

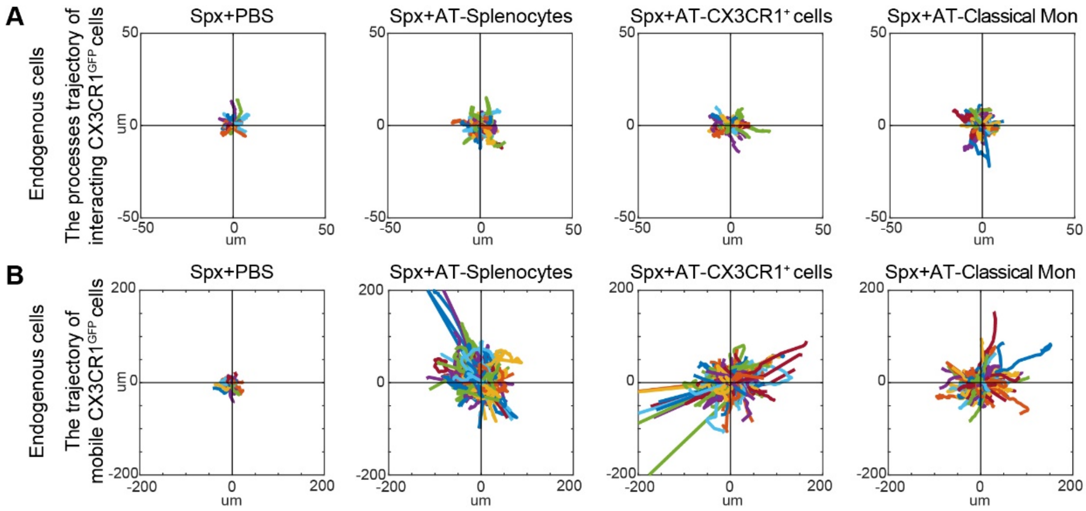


**Figure S15. Intravital imaging revealed the migration behavior of hepatic endogenous CX3CR1$^{GFP}$ cells in the fibrotic liver after adoptively transferring splenic cells.** (**A**) The processes trajectory of interacting CX3CR1$^{GFP}$ cells in the liver parenchyma. (**B**) The trajectory of mobile CX3CR1$^{GFP}$ cells in the hepatic vessels.

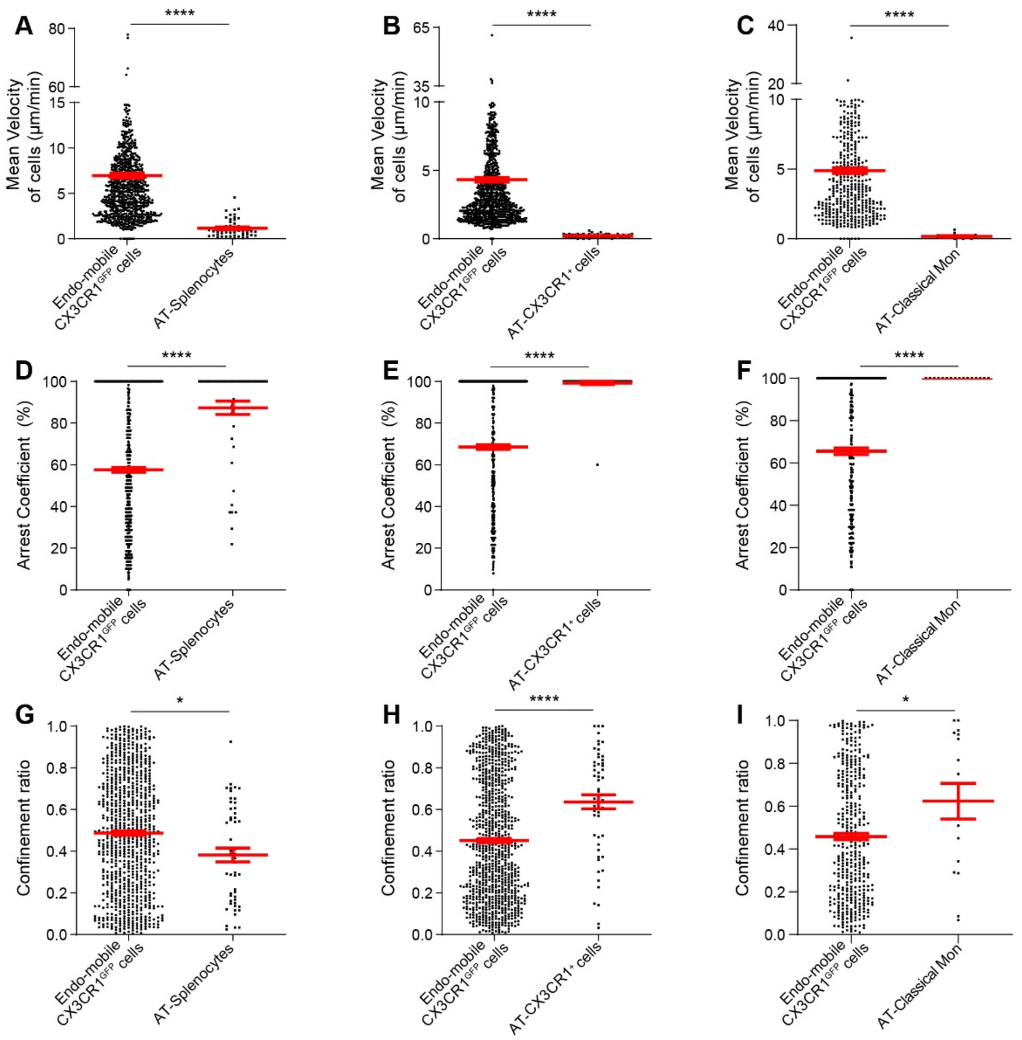


**Figure S16. Intravital imaging revealed the migration characteristics of hepatic endogenous CX3CR1$^{GFP}$ cells and adoptive splenic cells.** (**A-C**) Scatter plots of mean velocity of endo-mobile CX3CR1$^{GFP}$ cells and adoptive splenic cells in different groups (3 mice per group). (**D-F**) Scatter plots of arrest coefficient of endo-mobile CX3CR1$^{GFP}$ cells and adoptive splenic cells in different groups (3 mice per group). (**G-I**) Scatter plots of confinement ratio of endo-mobile CX3CR1$^{GFP}$ cells and adoptive splenic cells in different groups (3 mice per group).

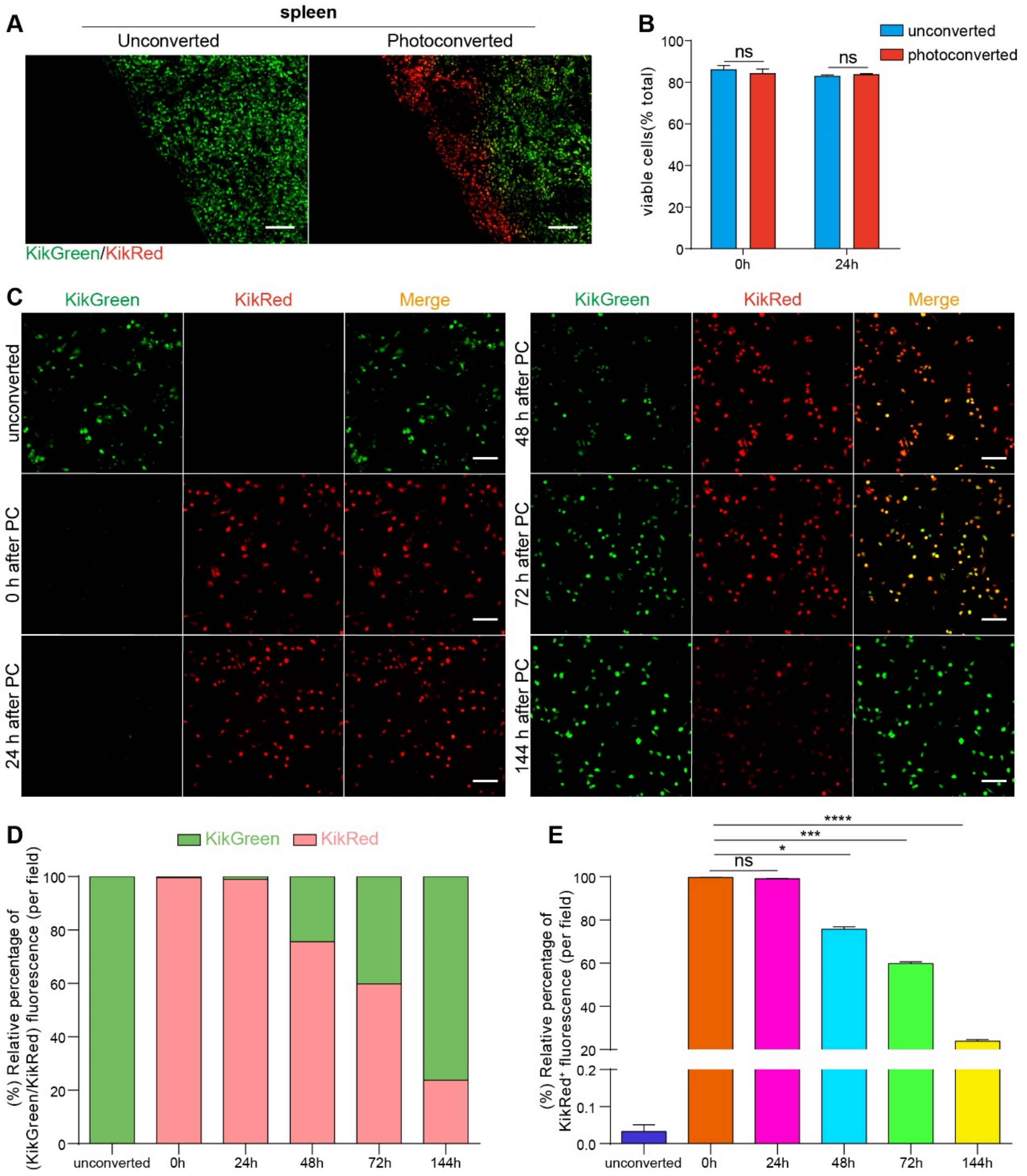


**Figure S17. The effect of the procedure of CX3CR1-KikGR photoconversion on cell viability and the stability of KikRed protein levels in CX3CR1$^{+}$ cells.** (**A**) Immunofluorescence imaging of unconverted and photoconverted spleen. Scale bar, 100 μm. (**B**) The viability of splenocytes from unconverted and photoconverted CX3CR1-KikGR transgenic mice was assessed using the fixable viability dye eFluor506 and analyzed by flow cytometry immediately (0 h) or 24 h after photoconversion (n = 3 mice per group). (**C**) Mouse peritoneal macrophages isolated from unconverted CX3CR1-KikGR transgenic mice were photoconverted by exposure to the 405 nm light (cells was exposed for 3 minutes, 200 mW/cm$^2$) *in vitro* and analyzed by confocal imaging immediately (0 h) or after culture for 24h, 48h, 72h and 144 h at 37 °C. Scale bar, 50 μm. (**D**) The

relative percentage of (KikGreen/KikRed) fluorescence of CX3CR1$^+$ mouse peritoneal macrophage was detected by confocal imaging. (n = 9 fields). (**E**) The relative percentage of KikRed$^+$ fluorescence of CX3CR1$^+$ mouse peritoneal macrophage was detected by confocal imaging. (n = 9 fields).

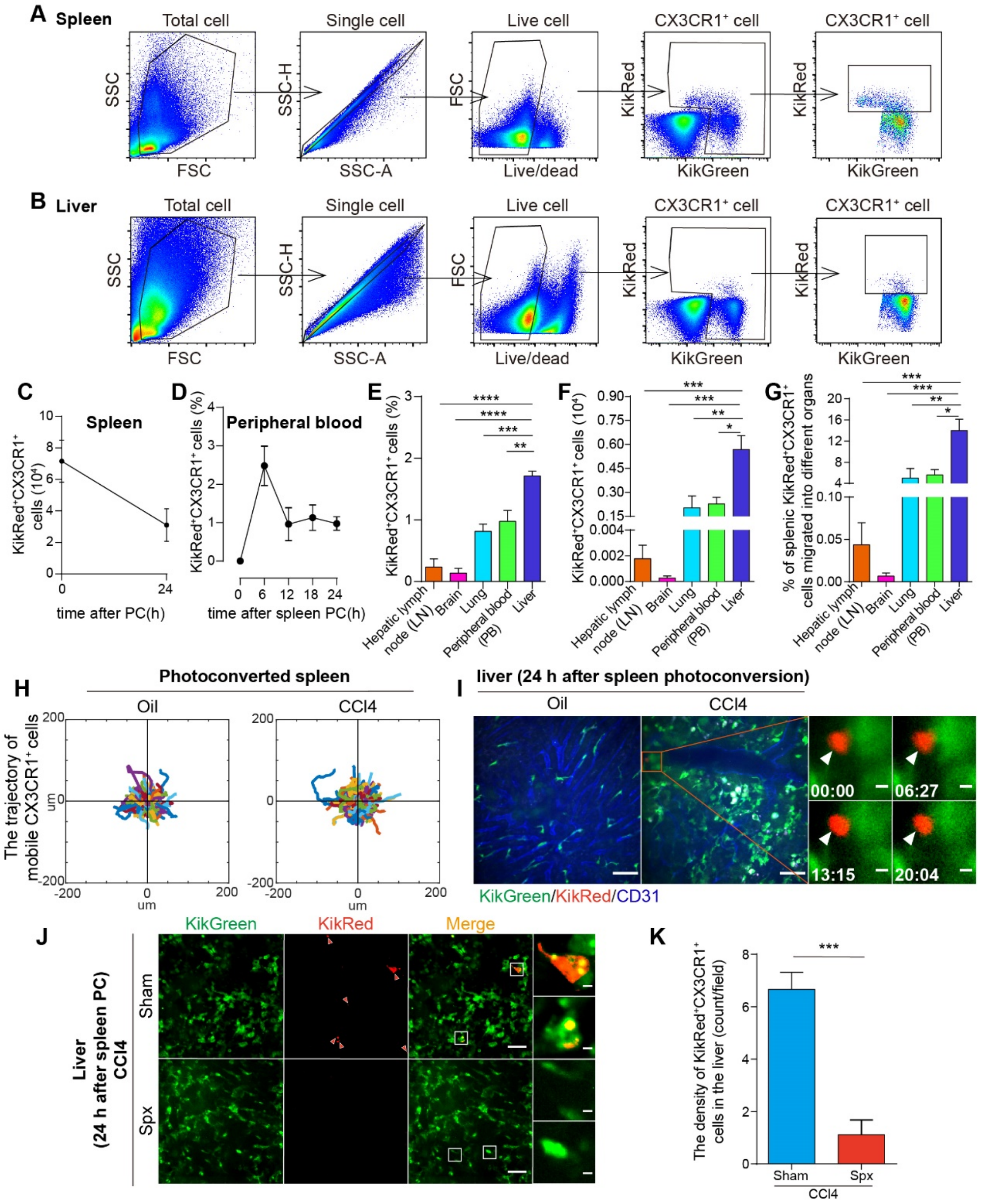


**Figure S18. Distinct dynamics of CX3CR1$^+$ KikRed$^+$ cells in the photoconverted spleen and liver from the Oil and CCl4-treated mice.** (**A**) Gating strategy of splenic CX3CR1$^+$ KikRed$^+$ cells. (**B**) Gating strategy of hepatic CX3CR1$^+$ KikRed$^+$ cells. (**C**) The number of photoconverted splenic CX3CR1$^+$ KikRed$^+$ cells egressed from the spleen (n = 4 mice per group). (**D**) The percentage of the CX3CR1$^+$ KikRed$^+$ cells among CX3CR1$^+$ cells in the peripheral blood over a 24-hour period (n = 3 mice per group). (**E**) The percentage of CX3CR1$^+$ KikRed$^+$ cells among CX3CR1$^+$ cells in different organs (lymph node, brain, lung, peripheral blood and liver) at 24 h after photoconversion (n = 3-5 mice per group). (**F**) The number of splenic CX3CR1$^+$ KikRed$^+$ cells in different organs

(lymph node, brain, lung, peripheral blood and liver) at 24 h after photoconversion (n = 3-5 mice per group). (**G**) The percentage of splenic CX3CR1$^{+}$ KikRed$^{+}$ cells migrated into different organs (lymph node, brain, lung, peripheral blood and liver) at 24 h after photoconversion (n = 3-5 mice per group). (**H**) The trajectory of mobile splenic CX3CR1$^{+}$ cells in the Oil and CCl4-treated mice. (**I**) Intravital imaging of the liver of Oil and CCl4-treated mice 24 h after spleen-specific photoconversion. Green: KikGreen; Red: KikRed; Blue: AF647 anti-CD31 labeled hepatic vessels. Scale bar, 50 µm. High-magnification views of splenic CX3CR1$^{+}$ KikRed$^{+}$ cells in the fibrotic liver are shown on the right. Scale bar, 5 µm. White arrows indicate splenic CX3CR1$^{+}$ KikRed$^{+}$ cells. (**J**) Intravital imaging of splenic CX3CR1$^{+}$ KikRed$^{+}$ cells in fibrotic livers of mice that received spleen-specific photoconversion at 24 h after splenectomy. Green: KikGreen; Red: KikRed. Scale bar, 50 µm. High-magnification views of splenic CX3CR1$^{+}$ KikRed$^{+}$ cells in the fibrotic liver are shown on the right. Scale bar, 5 µm. Red arrows indicate KikRed$^{+}$ cells. (**K**) The splenic CX3CR1$^{+}$ KikRed$^{+}$ cells in livers were counted (cells/field). (n = 9 fields, from 3 mice per group).

**Movie S1 and S2: Intravital imaging of CX3CR1$^{GFP}$ *cells in the livers of* Oil/CCl4-*treated mice at 24h after splenectomy.*** The time-lapse intravital imaging was acquired by a 20×/0.75 NA objective. CX3CR1$^{GFP}$ cells are shown in green, and the AF647 anti-CD31 labeled hepatic vessels are shown in red. Scale bar, 50 μm.

**Movie S3: Intravital imaging of CX3CR1$^{GFP}$ *cells in the livers of* CCl4-*treated mice at* 14d *after splenectomy.*** The time-lapse intravital imaging was acquired by a 20×/0.75 NA objective. CX3CR1$^{GFP}$ cells are shown in green, and the AF647 anti-CD31 labeled hepatic vessels are shown in red. Scale bar, 50 μm.

**Movie S4 and S5: Intravital imaging of hepatic CX3CR1$^{GFP}$ cells and adoptive splenic cells in the CCl4-treated mice with splenectomy at 24h after adoptive transferring of splenic cells.** The time-lapse intravital imaging was acquired by a 20×/0.75 NA objective. CX3CR1$^{GFP}$ cells are shown in green, adoptive splenic cells are shown in blue and the AF647 anti-CD31 labeled hepatic vessels are shown in red. Scale bar, 50 μm.

**Movie S6: Immunofluorescence imaging of hepatic CX3CR1$^{GFP}$ cells and splenic CX3CR1$^{+}$ cells in the CCl4-treated mice with splenectomy at 24h after adoptive transferring of splenic cells.** The immunofluorescence imaging was acquired by a 20×/0.75 NA objective. CX3CR1$^{GFP}$ cells are shown in green, splenic CX3CR1$^{+}$ cells are shown in blue and the AF647 anti-CD31 labeled hepatic vessels are shown in red. Scale bar, 10 μm.

**Movie S7: Immunofluorescence imaging of hepatic CX3CR1$^{GFP}$ cells and splenic classical monocytes in the CCl4-treated mice with splenectomy at 24h after adoptive transferring of splenic cells.** The immunofluorescence imaging was acquired by a 20×/0.75 NA objective. CX3CR1$^{GFP}$ cells are shown in green, splenic classical monocytes are shown in blue and the AF647 anti-CD31 labeled hepatic vessels are shown in red. Scale bar, 10 μm.

**Movie S8: Intravital imaging of splenic CX3CR1$^{+}$ *cells* in Oil/CCl4-treated mice after spleen photoconversion**. The time-lapse intravital imaging was acquired by a 20×/0.75 NA objective. CX3CR1$^{+}$ cells are shown in green, photoconverted CX3CR1$^{+}$ cells are shown in red and the AF647 anti-CD31 labeled splenic vessels are shown in blue. Scale bar, 50 μm.

**Movie S9: Intravital imaging of CX3CR1$^+$ KikRed$^+$ cells in the spleen of Oil/CCl4-treated mice after spleen photoconversion.** The time-lapse intravital imaging was acquired by a 20×/0.75 NA objective. CX3CR1$^+$ cells are shown in green, photoconverted CX3CR1$^+$ cells are shown in red and the AF647 anti-CD31 labeled splenic vessels are shown in blue. Scale bar, 20 μm.

**Movie S10: Intravital imaging of dynamic interaction between the splenic CX3CR1$^+$ KikRed$^+$ cells and hepatic CX3CR1$^+$ KikGreen$^+$ cells in the fibrotic liver at 24 h after photoconversion.** The time-lapse intravital imaging was acquired by a 20×/0.75 NA objective. CX3CR1$^+$ cells are shown in green, photoconverted splenic CX3CR1$^+$ cells are shown in red. Scale bar, 50 μm.

**Movie S11: Intravital imaging of stable interaction between the splenic CX3CR1$^+$KikRed$^+$ cells and hepatic CX3CR1$^+$ KikGreen$^+$ cells in the fibrotic liver at 24 h after photoconversion.** The time-lapse intravital imaging was acquired by a 20×/0.75 NA objective. CX3CR1$^+$ cells are shown in green, photoconverted splenic CX3CR1$^+$ cells are shown in red and the AF647 anti-CD31 labeled hepatic vessels are shown in blue. Scale bar, 50 μm.

**Supplementary Table 1. Primer sequences.**

| mRNA | Forward (5′–3′) | Reverse (5′–3′) |
|---|---|---|
| α-SMA | GTCCCAGACATCAGGGAGTAA | TCGGATACTTCAGCGTCAGGA |
| iNOS | GGAGTGACGGCAAACATGACT | TCGATGCACAACTGGGTGAAC |
| COL1A1 | GCTCCTCTTAGGGGCCACT | CCACGTCTCACCATTGGGG |
| IL-1β | GAAATGCCACCTTTTGACAGTG | TGGATGCTCTCATCAGGACAG |
| TNF-a | CCTGTAGCCCACGTCGTAG | GGGAGTAGACAAGGTACAACCC |
| Actin | GTGACGTTGACATCCGTAAAGA | GCCGGACTCATCGTACTCC |
